# Non-Tuberculous Mycobacteria: Molecular and Physiological Bases of Virulence and Adaptation to Ecological Niches

**DOI:** 10.3390/microorganisms8091380

**Published:** 2020-09-09

**Authors:** André C. Pereira, Beatriz Ramos, Ana C. Reis, Mónica V. Cunha

**Affiliations:** 1 Centre for Ecology, Evolution and Environmental Changes (cE3c), Faculdade de Ciências da Universidade de Lisboa, 1749-016 Lisboa, Portugal; andre.c.pereira94@gmail.com (A.C.P.); beatriz.g.ramos@gmail.com (B.R.); ana.reis714@gmail.com (A.C.R.); 2 Biosystems & Integrative Sciences Institute (BioISI), Faculdade de Ciências da Universidade de Lisboa, 1749-016 Lisboa, Portugal

**Keywords:** non-tuberculous mycobacteria, environmental mycobacteria, mycobacterial ecology, mycobacterial physiology, mycobacterial infection, mycobacterial diagnostics

## Abstract

Non-tuberculous mycobacteria (NTM) are paradigmatic colonizers of the total environment, circulating at the interfaces of the atmosphere, lithosphere, hydrosphere, biosphere, and anthroposphere. Their striking adaptive ecology on the interconnection of multiple spheres results from the combination of several biological features related to their exclusive hydrophobic and lipid-rich impermeable cell wall, transcriptional regulation signatures, biofilm phenotype, and symbiosis with protozoa. This unique blend of traits is reviewed in this work, with highlights to the prodigious plasticity and persistence hallmarks of NTM in a wide diversity of environments, from extreme natural milieus to microniches in the human body. Knowledge on the taxonomy, evolution, and functional diversity of NTM is updated, as well as the molecular and physiological bases for environmental adaptation, tolerance to xenobiotics, and infection biology in the human and non-human host. The complex interplay between individual, species-specific and ecological niche traits contributing to NTM resilience across ecosystems are also explored. This work hinges current understandings of NTM, approaching their biology and heterogeneity from several angles and reinforcing the complexity of these microorganisms often associated with a multiplicity of diseases, including pulmonary, soft-tissue, or milliary. In addition to emphasizing the cornerstones of knowledge involving these bacteria, we identify research gaps that need to be addressed, stressing out the need for decision-makers to recognize NTM infection as a public health issue that has to be tackled, especially when considering an increasingly susceptible elderly and immunocompromised population in developed countries, as well as in low- or middle-income countries, where NTM infections are still highly misdiagnosed and neglected.

## 1. Introduction: Mycobacteria and the Host

The *Mycobacterium* genus includes a high diversity of species, with differential phenotypic and genotypic traits, as well as epidemiological relevance, including several important human and animal pathogens [1]. *Mycobacterium* species can be included in three groups: (1) mycobacteria that cause tuberculosis (TB), (2) mycobacteria that cause leprosy, and (3) the non-tuberculous mycobacteria (NTM), a wider diverse group of species also referred in the literature as atypical or environmental mycobacteria [2]. Moreover, a differentiation according to host–pathogen relationships can be drawn, with pathogens capable to survive and replicate in the environment (free-living or facultative pathogens), while others cannot replicate outside the host (obligate or strict pathogens) [3]. *M. tuberculosis* and *M. leprae* are strict pathogens that together account for hundreds of thousands of new TB and leprae cases, respectively, and to a striking number of human deaths every year. Research interest is mainly focused on these two members, as well as on the animal-adapted TB ecotype, *M. bovis*, however, the number of reports of human and non-human infections caused by environmental or nosocomial NTM increased in the last decade, possibly resulting from global changes that impacted infection dynamics, better diagnostics, and/or increased awareness. The steady rise of the incidence of NTM infection has consolidated the attentiveness of academic and clinical communities, easing the identification of new cases and accelerating research in NTM-related topics [2].

NTM are opportunistic pathogens (i.e., microorganisms that can become pathogenic upon a perturbation of their host, such as a prior disease, co-infection, immunodeficiency, or aging) that can be found in natural and anthropogenic-related environments. The numbers of described NTM species have been increasing, posing challenges to their correct identification based on conventional methods that explore phenotypic traits and also the molecular approaches [4]. A wide variety of new methodologies have become available for the detection, identification, and differentiation of NTM species. These topics are reviewed further ahead.

The recognition of NTM as opportunistic pathogens began in the 1950’s with a number of patients suffering from underlying pulmonary disease being reported in the literature [5] Chronologically, three distinct phases could be drawn: in the pre-HIV era, the majority of patients with NTM pulmonary infection were men, whose risk factors included lung damage from other conditions (e.g., emphysema, cystic fibrosis, TB), smoking and occupational dust exposure [6], or World War II veterans with smoking and alcohol abuse habits; in a second stage, the infections became associated with patients with concomitant human immunodeficiency virus infection and loss of T-cells. Afterwards, reports describe taller, slender, and elderly women (and some men) as being more affected [7]. 

However, these bacteria only attracted more attention from the medical community when disseminated *Mycobacterium avium* complex (MAC) was recognized as one of the major opportunistic bacterial infections affecting acquired-immunodeficient syndrome (AIDS) patients [7]. After this acknowledgment, pulmonary infection in immunocompetent patients by NTM also became a concern, namely in developed countries, wherein trends for NTM infection have increased as pulmonary TB decreases [7]. Nowadays, the number of infection cases due to NTM almost outweighs those of TB in developed countries where NTM infection has been growing due to population aging, immunodeficiency, comorbidity with chronic diseases, and immunosuppressive therapy [8]. Contrary to TB, reporting of NTM infection is not mandatory, narrowing the epidemiological understanding of disease burden, especially in developing countries, where the prevalence of pulmonary TB infection is still high and NTM infection is poorly documented.

NTM infections are now known to be associated with a multiplicity of diseases, including pulmonary, soft-tissue, or milliary, however pulmonary infection is the major clinical presentation. The lymphatic system, skin, soft tissue, and bone/joints are also commonly affected [9].

Inside the host, mycobacteria are found within different cell types, but tropism to macrophages where mycobacteria are internalized into phagosomes and replicate has been highlighted by the vast majority of studies [10]. The arrest of the maturation of early mycobacterium-containing phagosomes and resistance to acidic and oxidative damage are well-known protective mechanisms of these pathogens [10]. The control of mycobacterial infection by the host relies on an immune response centered in CD4+ T cells that produce the macrophage-activating cytokine interferon-γ (IFN-γ) and also on tumor necrosis factor α (TNF-α) that is involved in maintaining the structure and integrity of granulomas [11]. While macrophage autophagy and apoptosis have been increasingly identified as important mechanisms for the control of intracellular pathogens, some NTM species can escape apoptotic bodies out to the extracellular space, infecting and spreading new cells and tissues [10].

Contemporary mycobacteria most probably emerged from ancestors adapted to terrestrial and marine environments that were selected for a unique cell wall and host speciation. The striking biological traits of mycobacteria foreordained by evolution currently distinguish NTM from any other microbes, being at the basis of their emergence as strict or opportunistic pathogens.

## 2. Mycobacteria Diversity and Evolution

### 2.1. General Characteristics of the Mycobacterium Genus

The genus *Mycobacterium* belongs to the *Mycobacteriaceae* family included in the *Actinobacteria* phylum and presently encompasses more than 220 recognized species and subspecies [1].

The genus was first proposed in 1896 by Lehmann and Neuman to include organisms described to be halfway between fungi and bacteria [2], and the first identified and studied members were *M. tuberculosis* and *M. leprae* [2].

Overall, the *Mycobacterium* genus includes aerobic to microaerophilic, slightly curved or straight rods (0.2–0.6 × 1.0–10 μm), non-motile, and asporogenous organisms [12]. The distinguishing characteristics of this genus include acid-fastness and the presence of mycolic acids. Although these are present in other Actinobacteria-related genera, only *Mycobacterium* species have long chains of mycolic acids with 60 to 90 carbon atoms [12]. This characteristic cell wall with lipid-rich content enables these bacteria to be differentiated based on staining techniques, once high mycolic acid content makes the organisms resistant to the decolorization with acid alcohol [12]. Therefore, mycobacteria are difficult to stain by Gram’s method, however, they are usually considered Gram-stain positive [12]. This genus includes organisms present in natural and anthropogenic-related environments, with high GC DNA content (ranging from 61 to 71%) and an optimal growth temperature ranging from 25 to 45 °C, depending on the species [12].

Mycobacterial genome sizes span a wide range, from 3.2 Mb (*Mycobacterium leprae*) to 8.0 Mb (*Mycobacterium mageritense*) [13]. Considering the growth rate, usually, mycobacteria are divided into two groups: the slow growers, that require more than 7 days to develop visible colonies on solid medium, and fast growers, that require 3–7 days to develop visible colonies. The colonies may be white- to cream-colored; however, some strains produce yellow to red-pigmented colonies with (i.e., photochromogenic) or without light (i.e., scotochromogenic) stimulation [12].

### 2.2. Phylogeny and Evolution of the Mycobacterium Genus

Within the genus *Mycobacterium*, both the growth rate and pigmentation of colonies are still commonly used phenotypic features to characterize different species, however, nowadays the exploitation of the molecular component overcomes the evaluation of phenotypic traits [4].

**Figure 1:**
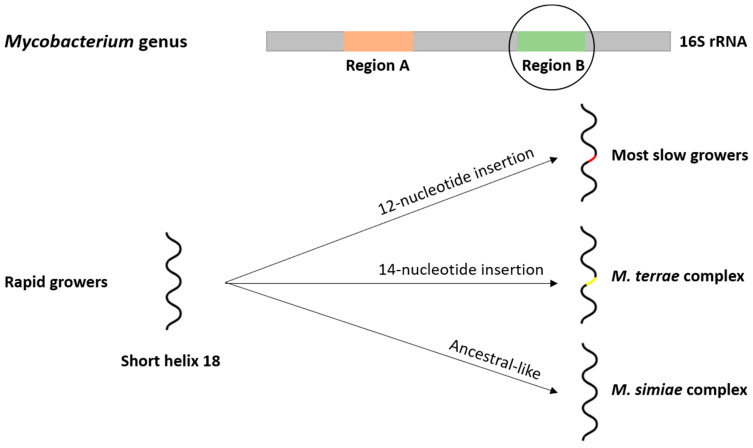
Evolution of the short helix 18 in the region B of the 16S rRNA gene. A 12-nucleotide insertion differentiates rapid-growing mycobacteria from slow-growing mycobacteria, except for *M. simiae* complex that shows an ancestral-like short helix 18 sequence and *M. terrae* complex that possesses a 14-nucleotide insertion, instead of the typical 12-nucleotide insertion.

The 16S rRNA encoding gene was, for many years, the basis of taxonomic works (Figure 1). Taxonomic and phylogenetic research were mainly focused on two hypervariable sequences known as regions A and B [14]. The 16S rRNA-based phylogeny endorses a division between rapid and slow growers, clustering most species in well-defined phylogenetic groups, however, the moderate variability of 16S rRNA among mycobacteria with high intraspecific identity (ranging from 94 to 100%) limits the distinction of several species [14]. Later on, the identification of a phylogenetic signature related to the short helix 18 in the hypervariable region B was reported, with most slow growers presenting a 12-nucleotide insertion that is not present in rapid growers [14]. Moreover, within the slow growers, two particular situations can be highlighted: (1) members of the *M. terrae* complex have a 14-nucleotide insertion; and (2) the members of the *M. simiae* complex present sequence features of short helix 18 that match those of ancestral mycobacteria [14].

The relationships among mycobacterial species and identification algorithms have also been evaluated over the years using the intergenic transcribed spacer region (between the genes encoding the 16S and 23S rRNA), and several housekeeping genes, including the 65-kDa heat shock protein (*hsp65*), DNA gyrase subunit B (*gyrB*), and RNA polymerase β-subunit (*rpoB*) [15,16]. These approaches include gene amplification by PCR, sequencing, restriction fragment length polymorphism analysis, and concatenated sequence analysis [15,16]. The phylogenetic reconstruction using the concatenated sequences of *hsp65* and *rpoB* genes supports the results obtained with 16S rRNA-based works [15,16].

The entry in the genomics era and the availability of whole-genome sequencing (WGS) platforms and bioinformatics pipelines enabled the revision of the *Mycobacterium* genus taxonomy. The first two WGS published works, based on a different number of genomes and methodologies, supported the same global conclusions: (1) a clear division between slow and fast growers and an intermediate position occupied by the *M. terrae* complex; (2) an ancestral position occupied by fast growers; (3) the members of *M. chelonae-abscessus* complex pointed out as the most ancestral cluster [17]. The analyses based on WGS data helped to highlight potential conflicts in the taxonomy, suggested new group assignment, and detected inconsistencies in the labelling of already available genomes [17].

A more recent work conducted by Gupta and collaborators (2018) performed comparative genomics analyses with 150 *Mycobacterium* genomes and gave a new perspective on the *Mycobacterium* genus. The phylogenomic tree based on core proteins, the high degree of genome relatedness among members of each clade, and the identification of specific molecular signatures, jointly supported the existence of five phylogenetic clades that were named “Tuberculosis-Simiae”, “Terrae”, “Triviale”, “Fortuitum-Vaccae”, and “Abscessus-Chelonae” clades [13]. The Abscessus-Chelonae clade was proposed as the earliest branching lineage within the *Mycobacterium* genus, as had been anticipated by previous works and supported the notion that slow growers evolved from fast growers [13]. Three of these clades comprise slow-growing species, while the other two clades mostly encompass the fast-growing [13]. This comparative genomics work allowed the identification of 172 molecular markers that are unique to all *Mycobacterium* species or members of different clades, which in the future can be explored to improve diagnostic algorithms [13]. A new division for the genus *Mycobacterium* comprehending only the members of the Tuberculosis-Simiae clade was proposed. Moreover, the remaining clades would be transferred to four new genera, with the following proposed names, *Mycolicibacter* gen. nov. (Terrae clade), *Mycolicibacillus* gen. nov. (Triviale clade), *Mycolicibacterium* gen. nov. (Fortuitum-Vaccae clade), and *Mycobacteroides* gen. nov. (Abscessus-Chelonae clade) [13].

### 2.3. Evolutionary Drivers of the Mycobacterium Genus

The increasing availability of data from genomics approaches brought insights into the evolutionary processes of *Mycobacterium* genus, however, most works remain focused on specific species or complexes, namely those of high clinical importance, such as the *Mycobacterium tuberculosis* complex (MTC).

Pan-genome analyses accomplished by different research groups underlined an open pan-genome [17,18], with the work conducted by Fedrizzi and collaborators (2017) identifying 150 thousand unique gene families that reflect the potential functional repertoire of mycobacteria [17]. Another work studied the balance between gene gain and loss and revealed that slow growers had generally gained and lost more genes compared to the rapid growers. The loss by slow growers of genes responsible for the access to extracellular nutrients was attributed to their slow growth rate [18]. The number of species-specific genes and relative gene balance might have contributed to mycobacteria adaptation to different ecological niches and environments and the differential pathogenic capacities of some members. 

Other works evaluate the importance of phenomena like horizontal gene transfer (HGT) to *Mycobacterium* genus evolution, being plasmid exchange one of those mechanisms. However, the majority of works focus a limited diversity of mycobacterial species that include MAC, *M. scrofulaceum*, and *M. fortuitum* [19]. Hybridization works revealed the relatedness of plasmids from *M. avium*, *M. intracellulare*, and *M. scrofulaceum*, isolated from clinical samples and the environment [20], suggesting that those plasmids had moved across species in the environment. Moreover, work conducted with *M. avium* and *M. intracellulare* show that plasmids occur more frequently among clinical isolates than environmental strains, suggesting a role in pathogenicity [21].

Experimental procedures have demonstrated that plasmids can be transformed and maintained into other mycobacteria, such as *M. tuberculosis* and *M. smegmatis*, indicating that the different environmental niches occupied by their hosts and the physical inability to spread might explain their limited host range [22,23].

The evolution within the *Mycobacterium* genus was traditionally considered clonal, however recent studies show evidence of HGT events in *Mycobacterium* genomes, particularly involving genes related with nutrient transport, metabolic processes, resistance against drugs, and defense mechanisms, that all together may have contributed to metabolic versatility, ecological adaptation, pathogenicity, and survival under harsh conditions [17,24,25]. In fact, plasmids isolated from *M. marinum* and *M. abscessus* contain mercury resistance genes [26,27], plasmids from *M. scrofulaceum* are associated with both mercury and copper resistance [28,29], and plasmids from *M. ulcerans* contain genes involved in toxin production [30].

## 3. Mycobacteria Ecology: The Underlying Resilience Biology

### 3.1. Genetic Variability

Mycobacteria strains recovered from the same clinical or environmental samples are mostly clonal, however, clonal variation appears to be frequent, as the recovery of mycobacteria isolates from the same sample that shows intraspecific variability in DNA fingerprinting and antimicrobial susceptibility profiles is recurrent [31,32]. Thus, cautionary evaluation needs to take place when antimicrobial susceptibility tests (AST) are being interpreted, since minimal inhibitory concentrations may vary across clonal variants. Thus, an analysis of several colonies should be performed in diagnostic, AST, and genotyping approaches.

Clonal variation was considered the most probable source of genetic variation in mycobacteria before the beginning of the genomics era. The wide genomic diversity of mycobacteria then became explained simultaneously by other phenomena, like plasmid-mediated horizontal gene transfer, transposition, and recombination, among other singularities. 

The processes of horizontal gene transfer mediated by plasmids are scarcely known in MTC and NTM in general. One of the main reasons is that, for members of MTC, as well as for *M. smegmatis*, there has been little effort regarding the characterization of mycobacterial plasmids [33]. In contrast, for MAC, *M. intracellulare*, and *M. fortuitum* some reports are describing a few plasmids hypothesized to be mobilized across species in the environment [33]. Previous studies isolated pAL5000 [34] and pJAZ38 [35] from *M. fortuitum*, pMSC262 [36] from *M. scrofulaceum*, and pVT2 [23] and pLR7 [37] from *M. avium*. However, most plasmids found in environmental mycobacteria lack the transfer-associated genes, e.g., *M. avium* plasmid pVT2 sequence revealed homology with some relaxases responsible for inducing a strand-specific nick in the origin of transfer, but simultaneously lack other conjugation-like transfer genes [23]. This can also mean that pVT2 requires a conjugative plasmid encoding the necessary genes to complete the transfer [23]. Furthermore, a plasmid from an epidemic strain of *M. abscessus* was sequenced and identified as a broad-host-range IncP plasmid, often found among Gram-negative bacteria [38]. This finding in a particularly epidemic strain may suggest that restriction in the host range might be caused by some inability to mediate the transfer of mobile elements across mycobacteria [38]. More recently, comparative genomics has enabled the recognition of similar genomic islands across the genomes of several NTM [25]. Clustering analysis identified a group including *M. marinum*, *M. ulcerans*, *M. abscessus*, and *M. smegmatis* that share common mobile genetic elements and almost identical plasmids [25]. Similarly, a study from Ummels and coworkers (2014) identified a large conjugative plasmid designated pRAW in *M. marinum*, with homologues in a set of other slow-growing NTM, namely *M. avium* subsp. *hominissuis*, *M. kansasii*, and *M. yongonense*. These plasmids appear to be efficiently exchanged between slow-growing species but no exchange has been demonstrated within fast-growing species, although authors also describe the presence of weaker homologues in the latter [39].

### 3.2. Transcriptional Regulation

The transcriptional network of mycobacteria is frequently referred to be composed by a large set of regulators, namely sigma (σ) factors that articulate within a distinctive transcriptome landscape and are re-orchestrated by a non-coding transcriptome. Among mycobacteria, the number of sigma protein genes in obligate pathogens is lower than in environmental opportunistic mycobacteria, as the panoply of conditions to which the latter have to adapt require the fine-tuning of gene expression and refined articulation within these sigma factors. In the obligate pathogen *M. tuberculosis* genome, 13 σ subunits regulate transcription upon specific in vitro and in vivo conditions, reprogramming mycobacterial metabolism and physiology. The housekeeping regulation is assured by two σ factors (σ^A^ and σ^B^), with the remaining 11 factors being recruited to respond to particular environmental conditions (σ^C^-^M^) [40,41]. Such factors belong to the σ^70^ family, whose members recognize two sequences in the promoter region of their target genes, the -10 element and the -35 element [40,41]. For *M. tuberculosis*, the first element is recognized as being much more conserved than the latter. Several studies have been developed to infer the role of each factor, either by expression profiling or more recently by the construction of deletion mutants. In addition to its function as a housekeeping regulator, the σ^B^ subunit was indicated as a major stress response factor [40,41]. The σ^B^, σ^F^, σ^G^, σ^I^, and σ^J^ were shown to be involved in stationary phase regulation [40,41]. Starvation was shown to be regulated by σ^D^, σ^E^, and σ^F^, while σ^E^ was also found to be involved during pH stress [40,41]. Growth under low-temperature reveals the involvement of σ^H^ and σ^I^, while under high-temperatures σ^H^, σ^E^, and σ^M^ play major roles in the regulation circuitry [40,41]. The σ^C^, σ^E^, σ^H^, and σ^J^ were reported to be convoluted in regulation under oxidative stress [40,41]. The existence of such a wide set of σ regulators enables a remarkable transcriptional adaptation to several environmental conditions. Comparative genomics studies have been employed to understand regulation across NTM members. Waagmeester and coworkers (2005) predicted the existence of 26 σ factors in the genome of *M. smegmatis*, twice as much as that found in the *M. tuberculosis* genome [42]. Although no orthologs of *sigC, sigI*, and *sigK* were found, a significant enrichment of *sigH* was uncovered and, to a lesser extent, of the *sigJ* and *sigL* subfamilies [42]. These paralogous members provide evidence of gene duplication and speciation in mycobacteria. Especially in the case of *sigH*, which is associated with oxidative and temperature stress, it may reflect the evolution of complex regulatory pathways developed in this saprophyte [42]. Similarly to *M. tuberculosis*, the roles of σ^A^ and σ^B^ housekeeping regulators in *M. smegmatis* and *M. abscessus* have been clarified, with evidence of co-transcription of housekeeping genes during exponential growth [43]. In *M. smegmatis*, *sigF*, a stationary phase regulator in *M. tuberculosis*, was also shown to be involved in heat shock, acidic pH, and oxidative stress responses [44]. 

In the fastidious *Mycobacterium avium* subsp. *paratuberculosis* (MAP), 19 putative σ factors have been identified across its genome [45], of which five are considered species-specific, one is homologous to *M. tuberculosis’*, two are homologous to *M. smegmatis’* and 11 are homologous sigma factors from both species [45]. Additionally, in MAP, the role of *sigH* appears to be highly similar to the one reported for *M. tuberculosis* as shown through experiments with a deletion mutant [46]. The *sigH* was thus found to regulate transcription when the cells were exposed to diamide and heat shock [46]. Among MAC members, *sigC* was found to be fused with *Rv0093c* (with an anti-σ factor signature) to yield a single protein, however, its function is yet to be uncovered [47].

Different strains of *M. marinum* harbor different sigma factor numbers: 17 σ factors are reported for *M. marinum* M strain, while for *M. marinum* T CCUG 20998 strain the reported number is 18 [48]. The σ^B^ and σ^E^ were also found to account for 80% of the σ factor transcripts of *M. marinum* T CCUG 20998 strain on the stationary phase, while on the exponential phase σ^A^ accounted for almost a quarter of σ factor transcripts [48]. This study also showed strain-dependent variation in the mRNA levels of different σ factors [48]. Furthermore, the authors reported increases in the mRNA levels of specific σ factors, such as *sigC* under the exposure to nitrous stress, *sigH* and *sigG* under the action of mitomycin C, *sigE* to osmotic stress, and *sigB* to acidic stress [48].

For the *M. chelonae-abscessus* complex, the reported number of σ factors is 17 to 19 [49]. Behra and coworkers (2019) reported three *sigJ* orthologs in the *M. chelonae* genome and four in the *M. abscessus* genome [49], while the absence of *sigK* in the *M. abscessus* genome was also registered [49]. 

### 3.3. Mycobacterial Outer Membrane

The ecology of NTM results from several biological features, namely slow growth, with the considered rapidly-growing mycobacteria possessing a slower growth rate than the majority of environmental bacteria from other genera; a hydrophobic and lipid-rich impermeable envelope; biofilm formation abilities; resistance to extreme pH stress; survival under anoxic or anaerobic conditions (e.g., both *M. avium* and *M. intracellulare* showed similar growth rates under 6% or 21% oxygen [50]); and a remarkable metabolic activity of recalcitrant carbon compounds.

The highly hydrophobic and impermeable cell wall of mycobacterial cells is the main structural characteristic responsible for the intrinsic ability of NTM to resist and persist in extreme environments. 

**Figure 2:**
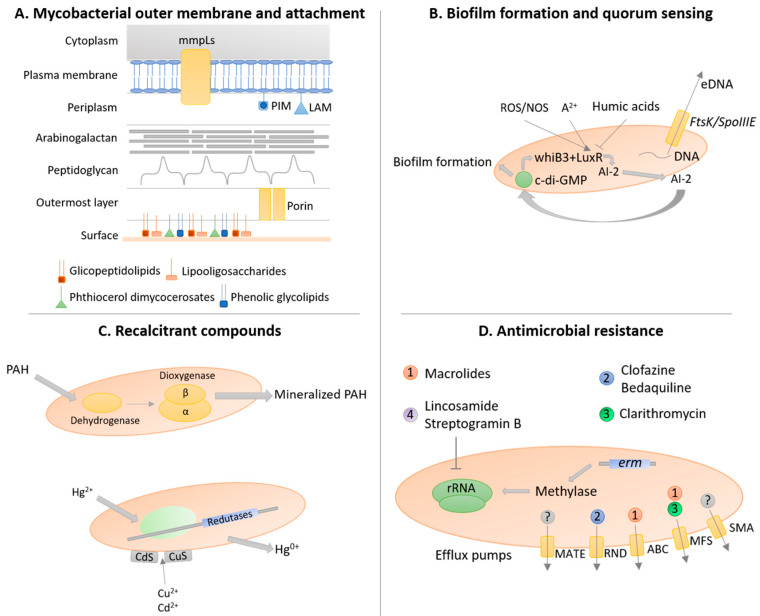
The resilient biology of non-tuberculous mycobacteria. (**A**) Non-tuberculous mycobacteria (NTM) possess a unique outer membrane with several biolayers that increase cell hydrophobicity and environmental resistance. In particular, several classes of lipids are extremely important for mycobacteria sliding motility and cell attachment to the surface, triggering early biofilm formation. (**B**) Biofilm formation is quorum-sensing dependent with oxidative stress (ROS/NOS) and divalent cations (A^2+^) promoting whiB3 and luxR gene expression, with the consequent increase production of autoinducer-2 (AI-2), leading to the increase in cyclic diguanylate (c-di-GMP) activation and biofilm formation-associated gene expression. Contrary, humic acids inhibit gene expression of biofilm formation-related genes. Additionally, eDNA is secreted into the extracellular matrix by FtsK/SpoIIIE secretion system. (**C**) NTM are also resistant to recalcitrant compounds, such as polycyclic aromatic hydrocarbons (PAH) and heavy metals. The degradation of PAHs is achieved by a dioxygenase system composed of a dehydrogenase, the dioxygenase small (beta)-subunit, and the dioxygenase large (alpha)-subunit, while mercury (Hg^2+^) triggers the synthesis of mercuric reductases and copper (Cu^2+^) and cadmium (Cd^2+^) are chelated into the mycobacterial cell wall as sulfides. (**D**) NTM can resist antimicrobials by several mechanisms, including: the intrinsic cell wall provides a physical barrier towards the entrance of antimicrobials; the erm genes that cause the methylation of rRNA, resulting in resistance to macrolides, lincosamide, and streptogramin B; and the expression of efflux pumps of different superfamilies, namely multidrug and toxic compound extrusion (MATE), resistance-nodulation-cell division (RND), ATP-binding cassette (ABC), major facilitator (MFS), and small multidrug resistance (SMR), that actively secrete several antimicrobial compounds. The information present in this figure was combined from data reported by several studies regarding various non-tuberculous mycobacteria species.

The composition and structure of the mycobacterial outer membrane is a major determinant of the growth, physiological, ecological, and virulence traits of NTM. The cell wall of mycobacteria is composed of an outermost layer (OL), a giant tripartite complex (mAGP complex) composed of the mycomembrane (MM), arabinogalactan (AG), and peptidoglycan (PG), together with a periplasmic space (Figure 2A) [51].

The MM is composed mainly of mycolic acids, with a heterogeneous composition depending on the leaflet side [51]. The inner leaflet is composed of mycolic acids covalently linked to AG, which is consequently covalently linked to PG [51]. The mycoloyl chains of mycolic acids are intercalated in a zipper-like manner, with the long mycoloyl chains being folded in an ω-shape [52]. Fatty acids, the base of mycolic acids, are produced by two fatty acid synthases: FAS-I, encoded by the *fas* gene, and FAS-II. The first produces acyl-CoA, while the second produces β-ketoacyl-ACP (MtFabD and MtFabH) followed by fatty acid elongation and maturation (HadA, HadB, HadC, InhA, KasA, and KasB) [53]. The mycolic acids are then activated (*FadD32* and *Rv3801c* genes), condensed (*Pks13* and *Rv3800c* genes), and reduced (*CmrA* and *Rv2509* genes) [53]. The outer leaflet of the MM is composed of various lipids, namely phospholipids, trehalose mycolates, glycopeptidolipids, and lipoglycans [51].

The PG is composed of both *N*-acetylmuramic acid (Mur*N*Ac) and *N*-glycolylmuramic acid (Mur*N*Glyc) in most mycobacteria, with *M. leprae* showing a PG exclusively composed of Mur*N*Ac [54]. The general presence of Mur*N*Glyc increases mycobacterial resistance to lysozyme, with the *namH* gene encoding the hydroxylase involved in *N*-glycosylation so that the deletion of this gene leads to lysozyme sensitivity [55]. Around 10% of the MurNAc of PG are covalently linked to AG by the phosphotransferase of the LytR-CpsA-Psr family named Lcp1 (*Rv3267* gene) [56]. The glycosylated PG fragments induce the production of TNF-α, which induces the recruitment and activation of phagocytic cells, potentiating the ecological niche within the host [57]. Additionally, the amidation modification of D-isoglutamate and mDAP residues are also crucial for the functioning of PG transpeptidases and the modulation of PG hydrolysis, reducing the net negative charge of the cell wall, hampering the action of antimycobacterial compounds [54]. The PG of mycobacteria presents a higher percentage of cross-linked peptides (70–80% compared to 40–50% in *E. coli*), being 33% of those cross-links composed of DD-(or 4→3) bonds between D-Ala and *m*DAP, while 66% are LD-(or 3→3) bonds linking two *m*DAP residues, reaching 80% during the stationary phase of *M. tuberculosis* [58]. Thus, this bond could be preferentially selected in the stationary phase as being more resistant to external stresses, improving the survival of mycobacteria. Moreover, PG fragments are also involved in cell signaling, with Rpfs producing those PG fragments that bind to PknB, leading to the resuscitation of dormant mycobacteria [59]. Furthermore, the host immune system is designed to recognize PG and PG fragments, triggering the first-line response of defense against mycobacteria. *Toll*-like receptors are triggered by lipomannan and lipoarabinomannan present on the cell surface or endosome/lysosome membranes [60]. Thus, any changes in PG composition could hamper the immune system response of the host.

The mycobacterial cell envelope possesses porins that help the mycobacterial cell to import the nutrients necessary for their constitutive metabolism, with MspA from *M. smegmatis* being the most well-characterized [61]. *M. tuberculosis* also presents OmpA (expressed by the *Rv0899* gene), however, the mixed alpha/beta-structure in replacement of an exclusive β-barrel transmembrane channel brings doubts on its true porin function [62]. In addition to porins, several other outer membrane proteins have been putatively identified by in silico analysis of *M. tuberculosis* genomes, namely mycoloyl transferases (e.g., antigen 85) [63], Rpf proteins [64], the outer membrane channel protein CpnT [63], and the sphingomyelinase SpmT (Rv0888) [65].

Externally, the OL, or capsule in pathogenic mycobacteria, can possess several compositions. *M. leprae* and *M. lepraemurium* have a capsule composed of phenolic glycolipids and glycopeptidolipids, respectively [66]. *M. tuberculosis* complex members have a capsule composed mainly of polysaccharides (glucan and D-arabino-D-mannan), a small fraction of glycolipids (2–3%), and a complex mixture of polypeptides [67]. Rapid grower mycobacteria, such as *M. phlei* and *M. smegmatis*, possess an OL composed mainly by a complex mixture of proteins [68]. 

### 3.4. Slow Growth

The characteristic slow growth and the underlying metabolism of NTM enable adaptation to change conditions, turning them less susceptible to antimicrobial agents and disinfectants [31,69] and/or to environmental stresses such as anaerobiosis [70], starvation [71], low pH [72], high temperature [73], and osmotic stress [74]. The general adaptation of mycobacteria to extremely stressful conditions is characterized by the entrance of the cell into a dormant state, slowing the metabolism and protecting the cell from life-threatening conditions [75]. The slow growth of NTM arrives from the lower number of 16S rRNA cistrons (one in slow growers or two in rapid growers) coupled with the division of resources for essential cellular processes, namely population growth that involves the high energy demanding process of long-chain C60-C80 mycolic acids synthesis that constitute the mycobacterial cell wall [76]. The diversion of energy (ATP) from making more cells to making a long chain lipid outer membrane is the major determinant of slow growth in mycobacteria. Additionally, the mycobacterial cell wall provides a barrier for stressors but also for nutrients, difficulting their entrance into the inner cell. In *M. smegmatis*, this problem is circumvented by the synthesis of porins, namely MspA, that has been shown to improve nutrient uptake and increase growth rate when over-synthetized [61]. This porin is also present in *M. fortuitum*, being related to increasing colony size, while it is absent in *M. tuberculosis* [77]. Moreover, it is possible that cell conditions, such as limited nucleotide pools, lead to restriction of new chromosomal rate synthesis since mycobacteria can multifork DNA replication and possess, at least in vitro, the DNA polymerase DnaE1 with a higher catalytic rate than that of *E. coli* PolIIIα [78].

### 3.5. Biofilm Formation and Quorum-Sensing

NTM have the natural ability to form biofilm-like structures, mainly due to their high cell surface hydrophobicity [51], dependent upon quorum-sensing-like mechanisms [79]. NTM spread onto solid surfaces by a sliding mechanism [80]. Sliding motility is mainly dependent on the existence of glycopeptidolipids that play a central role in biofilm formation in *M. avium*, *M. abscessus*, and *M. smegmatis* [81]. Hydrophobic interactions between fatty acid tails of the glycopeptidolipids of NTM and the hydrophobic solid surface enable attachment to surfaces and biofilm formation [80]. NTM are thus often considered as the first colonizers of mixed biofilm communities, then followed by other environmental bacteria. The species *M. abscessus*, *M. aurum, M. avium*, *M. gordonae*, *M. haemophilum*, *M. intracellulare*, *M. marinum*, *M. shimoidei*, *M. terrae*, and *M. ulcerans* have been recovered from water biofilm samples [82]. In *M. fortuitum* and *M. chelonae*, biofilm formation occurs under both high and low nutrient conditions [71].

In *M. abscessus*, deletion mutants of the *mmpL4b* and *mab_3168c* genes were shown to be impaired in biofilm formation and virulence [83,84]. In this species, there are two morphologically distinct phenotypes controlled by the product of the *mab_3168c* gene; when present, *M. abscessus* shows a rough colony type, characterized by corded microcolonies with higher tendency to form biofilms, and exhibits higher virulence; when the *mab_3168c* gene is absent, *M. abscessus* bacteria show a smooth colony type, characterized by smooth, rounded, small colonies with lower tendency to form biofilms, and a diminished virulence phenotype [84]. 

In *M. avium*, the ability to produce biofilms is associated with increased virulence and colonization of the bronchial mucosa [85]. In this species, biofilm formation only occurs in the presence of divalent cations (e.g., Ca^2+^, Mg^2+^, and Zn^2+^), and is enhanced in copiotrophic (e.g., higher presence of glucose and peptone) or oligotrophic environments, while it is inhibited in the presence of high concentrations of humic acids (Figure 2B) [86,87]. Additionally, exposure to oxidative stress leads to an increased release of autoinducer-2 that promotes biofilm formation, an adaptative strategy of mycobacteria to survive to extreme environments [88]. Another important component of biofilms is the presence of extracellular DNA that in *M. avium* is quorum-sensing dependent and excreted by the *FtsK/SpoIIIE* DNA transport system (Figure 2B) [89]. *M. avium* biofilm formation is also dependent on the activities of enzymes of the tricarboxylic acid (TCA) cycle (e.g., 6-oxodehydrogenase [SucA]), enzymes of glycopeptidolipid synthesis, a protein synthetase (pstB), and Rv1565c (a hypothetical membrane protein) [90]. Moreover, the biofilm formation of *M. avium* triggers TNF-α release that leads to apoptotic cell death of the macrophages, improving immune system evasion [91]. 

In *M. marinum*, cording of mycobacterial cells is commonly found and associated with biofilm formation, with lipooligosaccharides being the propellant for cell motility and biofilm formation [92]. Phthiocerol dimycocerosates and phenolic glycolipids are also crucial for cell-surface properties but not for cell motility, influencing biofilm formation [93].

In *M. ulcerans*, the extracellular matrix is composed of mycobacterial cell aggregates in discrete clusters; vesicles containing mycolactone and its biosynthetic machinery and more than 80 proteins with distinct roles in stress response and respiratory and intermediary metabolism; lipids (e.g., phosphatidylinositol mannosides, phospholipids, triacylglycerol, and phthiodiolone diphthioceranates); and carbohydrates (e.g., glucose) [94]. Mycolactone is a polyketide toxin that is considered a virulence factor responsible for dermonecrotic and immunosuppressive activities [94]. In addition to the production of these toxins, vesicles also protect mycobacterial cells from the antimycobacterial activity of antituberculous compounds [94]. Hsp18, a heat-shock chaperone, is hypothesized to play an important role in biofilm formation, namely in the cell adherence and attachment stages to surfaces [95].

**Figure 3:**
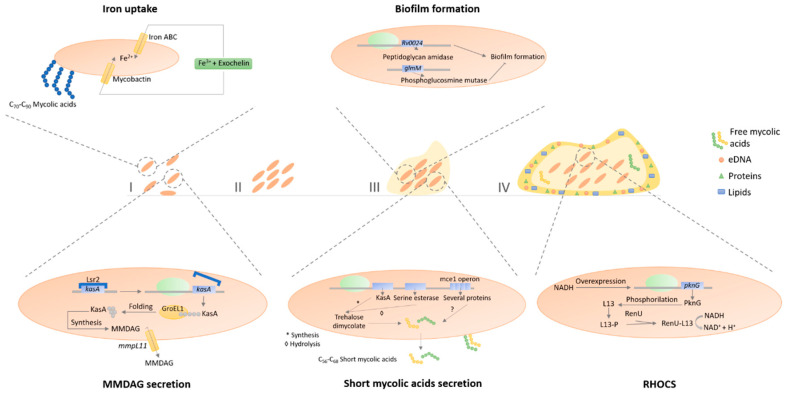
Biofilm formation of *Mycobacterium smegmatis*. *M. smegmatis* cells can be present in suspension on the environment (**I**), but under several environmental conditions, they aggregate in microcolonies (**II**), followed by an increase in cell density and formation of an extracellular matrix (**III**), leading to the formation of a mature biofilm (**IV**). In *M. smegmatis*, biofilm formation is iron (Fe^3+^)-dependent, producing siderophores (e.g., exochelin) responsible for chelating and transporting iron into the mycobacterial cell wall transporters (e.g., iron ATP-binding cassette (ABC) transporters or mycobactin). Monomeromycolyl-diacylglycerol (MMDAG) are important cell wall components involved in biofilm formation, with their secretion being dependent of Lsr2 and GroEL1 proteins regulation of the KasA protein synthesis and MMDAG secretion by mmpl11 efflux pump. Additionally, the *Rv0024* gene promotes biofilm formation, while the *glmM* gene inhibits it. Mycolic acids suffer a transformation from long mycolic acids (C_70_–C_90_) into short mycolic acids (C_56_–C_68_) when mycobacteria enter the biofilm state. The formation of those short mycolic acids is regulated by the ability of KasA to synthesize trehalose dimycolate, the ability of serine esterase to hydrolyze trehalose dimycolate into short mycolic acids, and by the mce1 operon. Additionally, short mycolic acids are secreted into the extracellular matrix. Mycobacteria cells in the biofilm phenotype have a reinforcing capacity to survive oxidative stress by expressing the redox homeostatic system (RHOCS). In this system, increasing concentration of intracellular NADH promotes the synthesis of PknG protein, which phosphorylates the L13 protein, increasing the binding capacity of L13 with RenU, leading to the oxidation of NADH into NAD^+^ and H^+^.

In *M. smegmatis*, mycolic acids are important cell wall components involved in pellicle biofilm, the type of biofilm formed in solid-air interfaces (Figure 3) [96]. The mycolic acids produced in late pellicle biofilms are shorter (C_56_–C_68_) than the ones produced during the planktonic lifestyle (C_70_–C_90_), being this mechanism regulated by the chaperone GroEL1 [96]. Moreover, free mycolic acids were also detected in the extracellular matrix as the result of the hydrolysis of trehalose dimycolate by serine esterase [96]. Furthermore, the *rpoZ* gene is also important in sliding motility and biofilm formation, since, compared to the wild type, deletion mutants have reduced levels of short-chain mycolic acids in the cell wall and absence of these in the extracellular matrix [97]. Monomeromycolyl diacylglycerol (MMDAG) and mycolate ester waxes present in the mycobacterial cell surface are important to form normal biofilms. Deletion mutants of the *mmpL11* gene, which encodes a transporter of these lipids, leads to a delay in biofilm formation [98]. Additionally, Lsr2 (histone-like protein) is also of crucial importance to the biofilm phenotype, since transposon insertion mutants in this gene exhibited deficiency in MMDAG, compromising biofilm formation through increasing sliding motility and by reducing cell surface hydrophobicity [99] which is essential for cell-to-cell interactions. In 2017, Yang and colleagues published a model of biofilm formation for *M. smegmatis* in which the planktonic cells start the aggregation processes by the overproduction of Lsr2 that further triggers the upregulation of GroEL1 and GroEL1-dependent free mycolate syntheses [100]. After this step, the iron sequestration pathways are induced, promoting biofilm maturation [100]. Additionally, *hadC* deletion mutants possess a defected dehydratase activity of fatty acid synthase type II (FAS-II), delaying, and attenuating pellicle biofilm formation [53]. The GroEL1 chaperone modulates the synthesis of mycolates specifically during biofilm formation and physically associates with KasA, a key component of the type II fatty acid synthase (FAS II) involved in mycolic acid synthesis [101]. The same occurs with mutants of the mammalian cell entry (*mce*) 1 operon, responsible for free mycolic acids accumulation on the mycobacterial cell wall [101]. Furthermore, *Rv0024* gene that encodes a putative peptidoglycan amidase induces biofilm formation by increasing cell hydrophobicity, contrary to the *glmM* gene that produces a phosphoglucosamine mutase, inhibiting biofilm formation [101]. Thus, the mycobacterial cell wall composition greatly affects the mycobacterial capacity to correctly form biofilms. Wolff et al. (2015) showed that biofilm formation is hampered by the absence of genes expressing the PknG, L13, and RenU proteins [102]. These proteins are part of the redox homeostatic system of mycobacteria (RHOCS) that detects the increase of NADH inside the mycobacterial cells resulting from redox stress situations, leading to the phosphorylation of the L13 protein by the PknG, increasing the association of L13 with the RenU protein, triggering the hydrolysis of NADH, then culminating in the redox homeostasis balance of mycobacterial cells [102]. In addition to this redox homeostatic system, thiol reductive stress is also implied in biofilm formation, with *mshC* and *mscR* genes (mycothiol biosynthesis and mycothiol dependent metabolism of nitrosothiols, respectively) being necessary to pellicle biofilm formation [103]. Thus, the metabolic status of mycobacterial cells regulates biofilm formation. Ojha et al. (2007) performed a transcriptional analysis of *M. smegmatis* cells in different stages of biofilm formation [104]. There was an overexpression of mycobactin biosynthesis genes, exochelin biosynthetic genes, and the putative iron ATP-binding cassette (ABC) transporter in the initial biofilm stages, showing iron uptake as a key aspect in *M. smegmatis* biofilm development [104].

Many trivial deficiencies of metabolism could affect biofilm formation by indirect means. For instance, metabolic deficiencies that directly or indirectly decrease the abundance and/or composition of cell wall components involved in the triggering, attachment, maturation, and/or maintenance stages of the biofilm structure may hamper the biofilm formation process and thus result in lower adaptability and survival capacity of NTM, indirectly causing lower virulence and pathogenicity in opportunistic infection scenarios.

Several intrinsic mechanisms of biofilm resistance to antimicrobials comprise physical or chemical diffusion barriers, slow bacterial growth, general stress response activation, and the rise of a biofilm-specific phenotype [105]. NTM cells grown in biofilms are transiently more resistant to disinfectants [69] and antibiotics [31]. Antibiofilm molecules, such as N-acetylcysteine and Tween 80, can be used to increase the antimicrobial effect of molecules against NTM biofilms. Biofilm environments also facilitate HGT between mycobacterial cells, since increased genetic competence, accumulation of genetic elements (e.g., antibiotic resistance genes), and high cell densities occur in those environments [106]. Conjugation is, until now, the only known mechanism for horizontal transfer of resistance genes in biofilms [106]. Moreover, a study in *M. smegmatis* showed that the recipient strain is incapable of forming biofilms, needing to be actively recruited to the biofilm structure by the DNA donor strain for conjugation to occur [106].

Mycobacteria possess a quorum-sensing-like mechanism, up to now described as being mainly involved in biofilm formation, however, the majority of accumulated knowledge on this topic is based on indirect research (e.g., bioinformatic analyses) and molecular and physiological research in *M. tuberculosis*. Among the known genes involved in the quorum-sensing mechanism, *LuxR* homologs have been previously detected in *M. tuberculosis* through bioinformatic analyses (Figure 2B) [107]. In addition to the detection of *LuxR* homologs in this species, several other homologs have been reported across multiple mycobacterial species, suggesting a common ancestry of quorum-sensing mechanisms in this genus [108]. Moreover, the *whiB3* gene, a putative transcriptional regulator whose differential expression has been shown to influence *M. tuberculosis* bacterial density during in vitro infection, has been pointed out as a regulator of quorum sensing (Figure 2B) [109]. Furthermore, quorum-sensing-dependent phenomena are usually regulated by second messengers that are responsible for signal transduction through phosphorylation cascades in the presence of autoinducers, the molecules segregated by bacteria to control response regulators activity [108]. A variety of molecules assumed as intracellular signaling players in mycobacteria species have been characterized, namely cyclic adenosine monophosphate (cAMP), cyclic guanosine monophosphate (cGMP), guanosine tetraphosphate ((p)ppGpp), cyclic diguanylate (c-di-GMP,) and cyclic diadenylate (c-di-AMP) (Figure 2B) [79]. These second messengers are known to regulate different phenotypes in bacteria at different stages of cell density and physiological states, as well as virulence and biofilm formation, so their expression in mycobacteria points to the existence of quorum-sensing circuitries. The (p)ppGpp is synthesized by the *relA* gene, while the c-di-GMP is synthesized by the *dcpA* gene; the absence of both genes leads to compromised biofilm formation, while the low level of these messengers triggers planktonic behavior, contrarily to high concentrations that trigger biofilm formation [110].

Mycobacterial biofilms are biological platforms that enable the colonization of environmental surfaces and a phenotype that overcomes the *status quo* of planktonic state due to the increased resistance of biofilms to environmental stress. Those structures are particularly found to be formed across water systems, both natural and artificial, as discussed ahead.

### 3.6. Resistance and Degradation of Recalcitrant Compounds

NTM are seldom auxotrophic, although fatty acid auxotrophy may occur sporadically. *M. avium*, *M. fortuitum, M. chelonae*, *M. intracellulare*, and *M. scrofulaceum* are oligotrophic [71,111]. The growth of *M. chelonae* and *M. fortuitum* species in commercial sterile distilled water at 25 °C has been described, with a slow decline of total viable counts throughout one-year, attributed to the ability of these mycobacteria to use trace amounts of volatile or micronutrients [112]. NTM are also capable of metabolizing recalcitrant compounds acting as environmental pollutants or carcinogenic substances, such as polycyclic aromatic hydrocarbons (PAHs) (e.g., phenanthrene, naphthalene, fluorine, fluoranthene, anthracene, pyrene, benzo[a]anthracene, benzo[a]pyrene), nitrogen-containing heterocycles (e.g., morpholine), polymers (e.g., vinyl chloride), alkanes (e.g., propane), humic and fulvic acids, and sterols (e.g., cholesterol) [113,114].

NTM are well associated with the metabolization of high molecular weight PAHs. Physiologically, they are adapted to multiply in highly concentrated PAHs environments due to different factors: (1) their hydrophobic envelope enables the transport of hydrocarbons without the need to synthesize surfactants since mycolic acids work as a biosurfactant that can be induced in response to hydrophobic substrate contact [115,116,117]; (2) the strong bound of PAHs to organic soil particles in oligotrophic conditions and oxygen depletion favors the proliferation of NTM [115,116,117]; 3) NTM biofilm formation on those soil particles enable higher accessibility to PAHs compounds [115,116,117]. 

For example, *M. vanbaalenii* PYR-1 has been one of the most studied strains regarding PAHs degradation (Figure 2C). It was first isolated in 1988 from soil persistently exposed to PAHs. This strain holds the capacity to mineralize naphthalene, phenanthrene, fluoranthene, pyrene, 1-nitropyrene, 3-methylcholanthrene, and 6-nitrochrysene into carbon dioxide [118]. The underlying degradation of PAHs is achieved by a dioxygenase system composed of a dehydrogenase, the dioxygenase small (beta)-subunit, and the dioxygenase large (alpha)-subunit [119]. *M. vanbaalenii* PYR-1 was also shown to degrade anthracene [120]. The metabolism of benzo[a]pyrene in this strain was also evaluated, with the initial oxidation being achieved by dioxygenases and monooxygenases. The stereo- and regioselectivity of the oxygenase involved in this pathway was confirmed [121]. *M. vanbaalenii* PYR-1 possesses *nidAB/nidA3B3* oxygenase that are involved in the degradation pathway of several PAHs, namely pyrene, fluoranthene, and phenanthrene, forming a phylogenetically distinct cluster from classical bacterial ring-hydroxylating oxygenase [122]. In 2006, a polyomic approach based on metabolic, genomic, and proteomic analyses was conducted to investigate the pyrene metabolism in *M. vanbaalenii* PYR-1 strain [123]. The results suggested the degradation of pyrene to central intermediates through o-phthalate and the β-ketoadipate pathway [123]. A year after, a similar approach was used to investigate the metabolic pathway of fluoranthene degradation in the same strain [124]. The results point to two main metabolic pathways, with fluoranthene being initially deoxygenated, followed by a pathway bifurcation: (1) degradation via fluorene-type metabolites; (2) oxidation via acenaphthylene-type metabolites [124]. Monooxygenation of fluoranthene can also occur as a detoxification reaction [124]. The comparative and functional genomic analysis of this strain led to the detection of two genomic regions where most of the 194 genes associated with the degradation of aromatic compounds are located, being the region A (150 kb long) the larger and with an atypical mosaic structure made of several gene clusters [125]. This strain genome analysis also revealed the presence of 28 genes involved in the TCA cycle, thus a pathway in which PAHs are degraded into the beta-ketoadipate pathway and then mineralized to carbon dioxide via the TCA cycle is likely to occur [125]. In 2011, an investigation of the metabolic network from *M. vanbaalenii* PYR-1 strain involved in PAHs metabolism was conducted [126]. The results highlight the scale-free architecture of the network with a funnel-like topology, in which many peripheral pathways converge to the β-ketoadipate pathway [126]. The central aromatic process is more conserved in evolution and function with its enzymes being relatively loosely regulated and functionally shared [126]. The ring cleavage process and the side chain process appear later in evolution, accomplishing their functions with relatively diverse specificity, with enzymes involved in the ring cleavage process being substrate-dependent and tightly regulated, while enzymes for the side-chain process are dispersed in modularity and redundant in function [126].

The cytochrome P450 gene families, that encode monooxygenases, are commonly associated with the degradation pathways of several compounds in NTM, including PAHs [127], sterols [128], alkanes [129], and morpholine [130].

NTM are also resistant to toxic heavy metals by compound degradation or compound sequester (Figure 2C). Mercury (Hg)-resistance in *M. scrofulaceum*, *M. marinum*, and *M. abscessus* is associated with plasmids carrying the metabolic operon for mercury-resistance [26,27,29]. *M. scrofulaceum* can reduce Hg^2+^ to insoluble Hg^0^ by means of a mercuric reductase that is rapidly lost from solution by volatilization [29] but also able to sequestrate cadmium (Cd^2+^) and copper (Cu^+2^) as sulfides in the cell envelope [29,131], that later are removed by precipitation. Both mechanisms protect mycobacteria but also other environmental bacteria cohabiting the same niche. Together with the metabolic capacity to degrade several toxic pollutants, such as anthracene and vinyl chloride, NTM are considered toxic waste dumps colonizers [113], degrading recalcitrant xenobiotics, detoxifying the habitats and enabling the colonization of other faster-growing microorganisms.

### 3.7. Antimicrobial Resistance

The outer membrane hydrophobicity and impenetrability of mycobacteria is the dominant key factor responsible for antimicrobial resistance and, thus, NTM are susceptible to a small fraction of known antibiotics only. They are intrinsically resistant to several antimicrobial agents, mostly the hydrophilic ones, as their hydrophobic outer membrane leads to low transport rates of hydrophilic compounds through the cell wall [76]. In parallel, the high mutation rate of the single 16S rRNA cistron, leads to the accumulation of resistance mutations towards ribosomal-targeting antibiotics [76]. Previous work has associated polymorphisms in the 16S rRNA encoding gene to amikacin resistance in *M. abscessus* [132]. Additionally, several other genomic mutations, plasmid transfer of resistance determinants, and the production of enzymes that metabolize drugs to a less active form have been described as resistance mechanisms [133,134]. Furthermore, the intracellular growth of NTM [10] combined with survival in caseum under a non-replicative state of bacterial persistence [70,135], mucus growth [135], and biofilm growth [136], make antibiotic delivery and action particularly difficult towards intrinsically resistant NTM [137]. Although all NTM possess some level of intrinsic resistance to antimicrobials, differences across NTM species have been demonstrated, with, for example, *M. kansasii* being susceptible to multiple antibiotics, while *M. avium* is susceptible mainly to macrolides [138].

The selection pressure that NTM are subjected to in natural environments, particularly in soil upon the action exerted by the antimicrobials (acting as selective agents) produced by their neighbors, has driven these bacteria to develop a panoply of resistance mechanisms to survive under these hostile environments [139]. In addition to the thick cell wall, NTM also possess efflux pumps that prevent the intracellular accumulation of drugs, therefore contributing to intrinsic and acquired resistance [140]. Efflux pumps belonging to the five superfamilies, namely ABC superfamily, major facilitator (MFS) superfamily, small multidrug resistance (SMR) superfamily, resistance-nodulation-cell division (RND) superfamily, and multidrug and toxic compound extrusion (MATE) superfamily were reported among NTM (Figure 2D) [140]. In MAC, ABC and MFS pumps are associated with macrolide resistance [141]; in *M. abscessus*, the RND pumps confer resistance to clofazimine and bedaquiline, while the overexpression of MFS pumps has been associated with clarithromycin resistance [142,143].

The ribosomal RNA methylase genes, including *erm*(38) in *M. smegmatis* [144], *erm*(39) in *M. fortuitum* [145], *erm*(40) in *M. mageritense* and *M. wolinskyi* [146], and *erm*(41) of *M. abscessus* and *M. bolletii* [147], have been commonly associated with intrinsic antibiotic-resistant phenotypes (Figure 2D). Moreover, in other mycobacteria, such as *M. boenickei*, *M. goodii*, *M. houstonense*, *M. neworleansense*, *M. porcinum, M. peregrinum*, and *M. wolinskyi*, the *erm* genes were also identified [146]. These genes are linked to two different phenotypes: high resistance to lincosamides and low to moderate resistance to macrolides and streptogramin B; or high resistance to lincosamides, macrolides, and streptogramin B, depending on the addition of a mono- or dimethyl group, respectively, to 23S rRNA, which decreases the binding of these antibiotics to ribosomes [148]. The expression of these genes can be induced upon drug exposure and a lack of correlation between phenotypic resistance and molecular detection of *erm* is also described [146]. Indeed, recent works suggest *erm* gene sequencing for accurate antibiotic susceptibility prediction [149].

Clinically acquired macrolide resistance in several mycobacteria, such as *M. avium* [150], *M. intracellulare* [151], *M. abscessus*, and *M. chelonae* [152]; or *embB* gene in *M. smegmatis* [153], has also been linked to polymorphisms in the 23S rRNA gene.

The treatment for NTM infection is therefore extremely long, difficult, and infection has a high probability of recrudescence. However, several alternative treatment approaches beyond conventional therapeutics, such as antimicrobial peptides, bacteriophages, iron chelators, or host-directed therapies, have recently been exploited in numerous studies, showing promising results.

### 3.8. Protozoa-Mycobacteria Symbiosis

**Figure 4:**
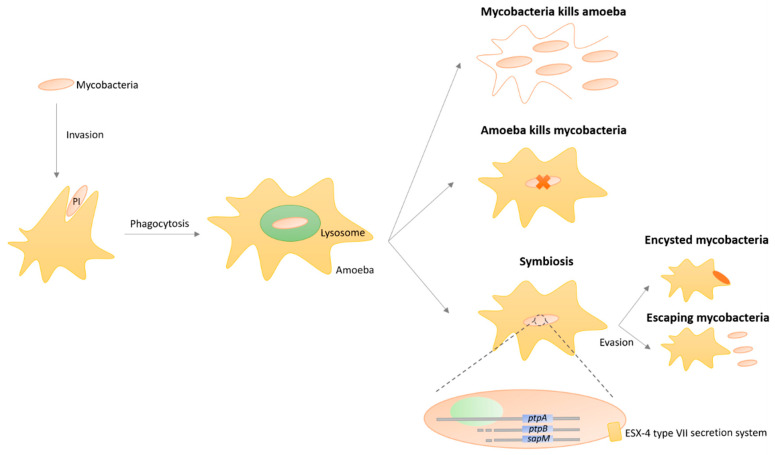
Mycobacteria–protozoa symbiosis. Pathogenicity islands (PI) promote mycobacteria invasion into amoeba, leading to phagocytosis. Three outcomes can result from this phagocytosis process: mycobacteria evade the lysosome and multiply, causing amoeba lysis; mycobacteria cannot evade the lysosome, causing mycobacteria death; or a symbiotic relationship occurs, with phosphatases encoded by the *ptpA*, *ptpB*, *sapM* genes being produced and excreted by the ESX-4 type VII secretion system. The symbiosis can lead to mycobacteria encystment or escapement from the amoeba. The information presented in this figure was combined from several studies regarding various non-tuberculous mycobacteria species.

Similarly to pathogenic mycobacteria and their ability to evade phagolysosomes and escape the lytic activity exerted by mammalian cells, namely the macrophages, NTM have the extraordinary ability to survive phagocytosis by protozoa such as amoebae and additionally grow as endosymbionts (Figure 4) [154,155,156,157,158]. NTM are preferentially predated by protozoa since these microorganisms graze biofilms, the usual niche of NTM in both natural and engineered environments [154,155,156,157,158]. Several works have highlighted the association of free-living amoebae with mycobacteria, with *M. leprae*, *M. avium*, *M. marinum*, *M. ulcerans*, *M. simiae*, *M. habane*, *M. gordonae*, *M kansasii*, and *M. xenopi* revealing the capacity of intra-amoebal survival inside vacuoles [159]. Work performed with *M. avium* suggests that genes organized in a pathogenicity island (PI) are connected to the bacterial capacity of invading amoeba, once mutants lacking the PI exhibited defects in *Acanthamoeba castellanii* invasion [160]. Moreover, *M. avium* persistence inside amoeba is associated with the up-regulation of several genes involved in transcription regulation, metabolic pathways, degradation of macromolecules, and membrane proteins, with a few being also related to the survival process inside macrophages [161].

The cell surface hydrophobicity and composition are known to play a crucial role in phagocytosis by amoeba and protozoa, including in pathogens other than mycobacteria [162]. Indeed, co-culture works developed with *M. marinum* and amoebae suggested the presence of anti-phagocytosis factors in the cell wall composition [163]. Furthermore, a recent work linked the action of three phosphatases (PtpA, PtpB, and SapM) to the mechanisms of intracellular replication of *M. marinum* in amoeba, revealing their combination to reduce vacuole acidification and to enable escaping the vacuole towards the host cytosol [164].

For *M. abscessus*, the ESX-4 type VII secretion system has been implicated in resistance to amoeba phagocytosis, with mutants in *esx-4* genes exhibiting a reduction in intra-amoeba replication [165]. Furthermore, a study analyzing the transcriptomic profile of *M. abcessus* in amoeba revealed a group of 45 up-regulated genes that allow mycobacteria to resist environmental stress and induce defense mechanisms [166].

The possibility to grow in endosymbiosis with protozoa protect NTM from extreme environmental conditions, namely the pressure exerted by natural antimicrobial compounds and starvation, especially due to NTM survival in encystment structures [154,155,156,157,158]. The persistence within amoeba has been related to the increase in the infectivity and virulence of *M. avium* and *M. abscessus* [167]. Additionally, this symbiotic relationship also benefits the phagocytic protozoa, for instance in the *Tetrahymena* spp. that acquire an evolutive advantage by ease access to lipids provided by intracellular NTM [168]. Although nowadays amoebae are recognized as an important reservoir [169], the underlying implications in the ecological, epidemiological, and public health patterns of NTM are unclear. Therefore, the historical importance of endosymbiosis with amoebae in NTM transmission, evolutionary selection of virulence traits, and adaptation to the macrophage needs further study. Interestingly, the acid fastness character of NTM is lost inside protozoa, indicating adaptative changes in the architecture of the intracellular NTM bacterium, mainly the outer membrane, supporting the hypothesis that protozoa may have played a fundamental role in the evolution of mycobacterial pathogenesis, selecting mycobacteria that can infect and replicate in protozoa, later becoming intracellular pathogens in animals [157].

## 4. Mycobacteria Ecological Niches

NTM are a cosmopolitan group of bacteria that can grow in a variety of natural and human-made environments, such as soils, water, dust, and air. Survival and replication in water-damaged building materials [170], cigarettes [171], metal-working fluid [172], ceramic products [170], and even several food products [173] have been reported. Additionally, associations of NTM with several aquatic invertebrates such as South China Sea sponges [174] and reef coral *Porites lutea* [175] have been described.

The geographical variation in environmental conditions leads to NTM species-specific variability in natural and human-engineered environments to which susceptible individuals are exposed [176]. A recent global soil survey revealed contrasting environmental preferences of mycobacteria, with associations between environmental factors (pH, aridity, and temperature) and their distribution, being MAC members more commonly detected in wet and acidic soils [177]. Similarly, the survival of mycobacteria in water resources was already studied, with works performed in water distribution systems, with the identification of *M. gordonae*, *M. kansasii*, *M. intracellulare* and *M. chelonae* in Portugal [178]; and *M. lentiflavum*, *M. tusciae*, *M. gordonae* in Finland [179], revealing a differential human exposure. Furthermore, a recent collaborative work based on the analysis of pulmonary clinical samples of individuals with NTM infections revealed their differential distribution by region [180].

### 4.1. Water

The ability of NTM to grow in water environments has been under the focus of numerous studies in natural sources, such as lakes, rivers, streams, and seawater [181], but also artificial environments, such as drinking water pipelines [182], hot tubs [183], foot-baths [184], residential faucets [185], hospital faucets and ice machines [186], diagnostic laboratories [187], bottled and municipal water [185], showerheads [31], brook waters [188], cooling towers [189], and wastewater treatment plants [190]. The concomitant presence of NTM and *Legionellae* in water distribution systems has been previously reported [191], often attributed to several biological similarities between both microbes.

Due to their hydrophobicity, NTM cells become attached to air bubbles that rise in the water column, reach the surface and burst, forming craters that collapse, leading to water droplets formation [192]. These droplets are enriched in NTM up to 10,000 times when compared with the water column, and can posteriorly be inhaled, reaching mammal lungs [192]. A great concentration of organic materials occurs at the air–water interface, together with the accumulation of NTM-containing bubbles, leading to a favorable environment for NTM enrichment [193]. The fact that NTM hold highly-hydrophobic cell walls facilitating aerosolization and surface adherence may explain their highly-infectious behavior.

In marine waters, NTM are present in lower burden due to the high concentration of NaCl, a growth inhibitor for most species [194]. Therefore, freshwater tanks show a greater burden of NTM than those tanks supplied with seawater [194].

NTM possess a natural capacity of colonizing and persisting in several human-engineered environments, such as water systems, being able to enter these systems through several routes, namely cross-connections and backflows, equipment or personnel before entry, finished water storage vessels, improper treatment of materials, inadequate distribution system security, joints and seals, leaking pipes, valves, and water treatment breakthroughs [195].

In surface water, NTM enter drinking water treatment systems by soil particle attachment, so the reduction of water turbidity leads to a reduction of NTM burden entering the water system [82]. Additionally, NTM naturally aggregate in water, a characteristic that favors increased resistance to disinfection in wastewater treatment plants, with inactivation being particularly compromised by aggregates larger than 41 µm [196]. Their intrinsic capacity to resist the action of several disinfectants, such as chlorine, contrary to the majority of other environmental bacteria, lead to a competitive advantage resulting in nutrient consumption and biofilm formation in the absence of competition, also reducing the likelihood of wash out [197]. NTM concentration increases in pipe systems far distant from the treatment plant [73]. In additoin, NTM cells growing in biofilms are more resistant to disinfection [31,69]. However, cells released from the biofilm would be transient of intermediately resistant to disinfectants and antibiotics, returning to similar susceptibility phenotypic profiles of suspension-grown cells [31,69]. Thus, the reduction of water particulate content reduces NTM burden in treated water. Nowadays, a shift from chlorine to chloramine has been occurring in the drinking water industry to avoid the production of halogenated carcinogens and to increase the microbicide effect over biofilms [191]. However, the frequency of recovery of NTM increased [191]. Further studies are thus needed to fully understand this issue and evaluate the public health impact of this measure. Moreover, households with lower water temperatures (<55 °C) are more likely to harbor NTM that those with higher water temperatures (>55 °C) [73]. *M. avium*, *M. intracellulare*, *M. kansasii*, and *M. xenopi* have been isolated from hot water systems (>50 °C) [195]. Additionally, slow sand filtration systems appear more efficient in NTM removal than rapid sand filtration, with NTM being able to colonize and grow on granular activated carbon and enter water distribution systems [198]. Furthermore, low dissolved-oxygen levels and elevated nutrient concentrations (nitrogen components and assimilable organic carbon), characteristic of eutrophic aquatic environments, showed a positive link with NTM burden [199].

### 4.2. Soil

In addition to water environments, the soil is also a frequent source of NTM, namely, potting soil [200] and coniferous forest soils [201]. NTM have been isolated from soil in multiple countries and climates, including Alpine habitats [202], Finland [201], Uganda [203], and Iran [204], among others [177].

The presence of NTM in the soil is strongly associated with several physicochemical characteristics, such as high content of humic and fulvic acids [205], sand-filtered water [198], low concentrations of oxygen [206], low pH [207], and high iron, zinc, and manganese concentrations [206,207]. Humic acids can be used as the unique carbon and energy source for mycobacteria within a biofilm [208]. The same features that account for the natural resistance of NTM to low concentrations of oxygen and low pH in the soil support their natural resilience inside the mammalian host [206]. The burden of NTM is higher in biofilms adherent to small particles, in particular the upper horizon where carbon availability is higher [209]. Furthermore, the cross-contamination between soil and water sources can occur by soil-dwelling NTM [206]. Permeability of soil is decreased during rainy seasons, which is beneficial for the survival and multiplication of NTM [206].

A recent study by Walsh and colleagues (2019) evaluated the diversity, distribution, and environmental preferences of mycobacterial species present in 143 soil samples collected from a range of different biomes [177]. Most detected mycobacteria clades (97%) were previously undescribed lineages, with a tendency to higher relative abundances being registered in cool, wet, and acidic soils [177].

### 4.3. Plants

NTM are frequently isolated from plants. *M. terramassiliense*, *M. rhizamassiliense*, and *M. numidiamassiliense* were first described in tomato plant roots [210] and *M. sarraceniae* and *M. helvum* were first isolated in the pitcher plant in Minnesota sphagnum peat bogs [211]. *M. avium* was detected in the stems and leaves of different plant species after internalization into plant tissue through intact, as well as damaged, root systems, which can imply the role of plants in the spread and transmission of mycobacteria to other organisms in the environment [212]. Moreover, several plant-based foods have been investigated and found to be contaminated with NTM, namely those that are close to, or beneath, the soil surface [213]. NTM can still be present in contaminated food after washing. Additionally, a previous study reported the same genotypes of *M. avium* in both infected patients and ingested contaminated food items [214]. Zwielehner et al. (2008) studied the lactic bacterial communities present in the phyllosphere of lettuce leaves and detected several *Mycobacterium* spp. sequences, mostly *M. alvei*, in both leaves and soil [215]. *M. scrofulaceum* is a frequent contaminant in the culture of plant tissue cell lines [216]. Furthermore, *M. ulcerans* have been reported to grow and form biofilms associated with aquatic plants, implicating these plants as reservoirs of *M. ulcerans* and establishing potential new links in the transmission chains involving humans [217]. Nevertheless, no clear proof of a real association (e.g., symbiotic, pathogenic, or mutualistic relation) between mycobacteria and plants has been demonstrated, with only co-isolation being confirmed.

### 4.4. Dust and Air

Since dust particles, namely peat-enrich dust, are commonly suspended in the air, inhalation of mycobacteria associated with dust particles is a plausible source of pulmonary infection [76]. In Germany, the abundance of *M. avium* subsp. *hominissuis* (Mah) was evaluated in several environmental samples, with 33% of the dust samples carrying this species [218]. In Korea, 5% of the analyzed air conditioner dust samples showed the presence of NTM [219]. Torvinen and colleagues (2010) developed a real-time PCR detection method to analyze the presence of NTM in house dust achieving a maximum burden of 7.2 × 10^6^ cell/g, with the majority of isolates belonging to *M. terrae* and *M. avium* complexes [220]. Leski et al. (2011) applied a broad-range resequencing array and detected the presence of fast-growing mycobacteria in desert dust samples from both Kuwait and Iraq [221].

The presence in air is also reported, mostly from aerosolized particles, namely shower aerosols [185] and hot tub aerosols [222]. In addition to household sources, aerosols generated in workplaces, gyms, public places (fountains), cooling towers on buildings, and rivers and streams (waterfalls) have also been indicated as possible sources for infection.

### 4.5. Extreme Environments

The physiological characteristics of mycobacteria referred to in the above topics show their extraordinary ability to live in extreme habitats. There are reports of NTM being found in caves, namely in sulfur caves in Romania [223]. These caves possess a unique environment where life can be found in a gas chemocline, with the atmospheric lower part of the cave being mainly composed of carbon dioxide (CO_2_), methane (CH_4_), and hydrogen sulfide (H_2_S), while the atmospheric upper part shows a similar composition to the atmospheric air found in Earth’s surface [223]. Additionally, both elemental sulfur (S^0^ and sulfuric acid (H_2_SO_4_) cover the cave walls resulting in a pH lower than 1. Therefore, all these extreme conditions (e.g., local anaerobiosis, high CO_2_ levels, extremely low pH, and absence of sunlight) make life in the cave almost impossible. However, strikingly, microbial biofilms can be detected in cave walls with NTM dominating among those communities [223], showing that NTM are capable to survive and multiplicate in niches with pH lower than 1, with limited availability of carbon and free energy sources, and with low humidity. The most likely source of energy in this environment is sulfur oxidation with H_2_S and S^0^ as reductants and atmospheric O_2_ as oxidant, however, ferric iron can also be a potential oxidant [223]. This was not the first time that NTM were reported as having chemolithoautotrophic growth. In the analysis of an Angkor monument sandstones, the ability of five NTM isolates to grow both chemolithoautotrophically using S^0^ and chemoorganoheterotrophically using organic substances was described [224]. Those isolates were phylogenetically related to *M. cosmeticum* and *M. pallens*. This autotrophic growth capacity with hydrogen [225] and the chemolithotrophic growth on carbon monoxide as the sole carbon and energy source under aerobic conditions have been reported previously in *M. flavescens*, *M. gastri*, *M. neoaurum*, *M. parafortuitum*, *M. peregrinum*, *M. phlei*, *M. smegmatis*, *M. tuberculosis*, and *M. vaccae* [226]. Additionally, the role of the assimilatory sulfur metabolism in virulence, antibiotic resistance, and antioxidant defense of *M. tuberculosis* has been examined [227]. 

NTM are also able to survive in extremely alkaline environments, with reports from Rio Grande, USA, where *M. fortuitum* complex, *M. scrofulaceum, M. semiae, M. gordonae*, MAC, *M. smegmatis*, *M. interjectium*, *M. lentiflavum*, *M. nonchromo*, *M. celatum*, *M. chitae*, *M. pheli*, and *M. kansasii* have been found [228]. 

The Yellowstone National Park possesses a variety of extreme environments that have been the target of research for several decades. A study focusing on the geobiology of a microbial endolithic community in the Yellowstone geothermal environment showed that 37% of the total bacterial rRNA detected belonged to *Mycobacterium* species [229]. Later, *M. parascrofulaceum* was isolated in Norris Geyser Basin, Yellowstone National Park, a system that possesses pH 3.0 and maximum temperatures of 56 °C [230]. *M. parascrofulaceum* was also found in hot springs from Zambia and Kenya with a pH range between 5 and 10 [231], suggesting that this species may be ubiquitous in hot springs, independently of the pH value.

Another recent study by Pavlik et al. (2018) investigated the occurrence of NTM in the extreme environment of the water zone of the Hranice Abyss, Czech Republic, the deepest flooded pit cave in the world [232]. This cave is characterized by its acidic water, high concentration of CO_2_, and the biofilm communities of slime bacteria. Interestingly, *M. arupense*, *M. avium*, *M. florentinum*, *M. gordonae*, *M. intracellulare, M. mucogenicum*, and *M. sediminis* were detected in this study.

Furthermore, *M. elephantis* was isolated from low impact rock surfaces [233], *M. gordonae* was identified in calcareous siltstone beds [234], and the *Mycobacterium* genus was also detected in Khuangcherapuk cave in India [235]. The overall presence of NTM species in cave oligotrophic environments has been associated with earthworm castings, bat guano, alluvial sediment, and plant material that provide NTM with the necessary organic matter [232].

Some NTM are thermotolerant, being able to optimally grow at 45 °C, such as *M. xenopi*, *M. thermoresistibile*, and *M. phlei*; however *M. hassiacum* is the most thermophilic mycobacteria, being able to grow at temperatures between 30 and 65 °C [236]. *M. hassiacum* is capable to cause infection in humans [237] but is usually found in water distribution systems [238]. The thermophilic behavior can be due to a down-regulation of trehalose biosynthesis at higher temperatures, high accumulation of free glucose as a potential carbon reserve for growth under more favorable conditions, and the thermostability of glucosyl-3-phosphoglycerate synthase responsible for the synthesis of the precursor of glucosylglycerate, a metabolite involved in mycobacterial survival under nitrogen starvation [236].

### 4.6. Healthcare Settings

NTM are also present in hospital environments, where contaminated materials can cause nosocomial outbreaks and pseudo-outbreaks [239]. Simple prevention measures such as avoidance of medical instruments’ and patient wounds’ exposure to tap water show a positive effect on the reduction of nosocomial infections by NTM [240]. The colonization of potable water systems by NTM in hospitals may cause pseudo-outbreaks which are by definition clusters of pseudo-infections, i.e., positive cultures from patients without evidence of infection or colonization, typically caused by environmental contamination during specimen handling [241].

Furthermore, dental unit waterlines are particular hotspots for NTM due to their capacity to rapidly develop biofilms on the dental water supply lines combined with the formation of potentially contaminated aerosols [242]. The biofilms are promoted by the plastic material used for tubing and the architecture of the machinery that leaves a thin layer of water almost undisturbed against the machinery walls and surfaces [242]. These waterlines possess a higher burden of both NTM, particularly *M. chelonae*, *M. flavescens*, *M. gordonae*, *M. simiae*, and protozoa, namely amoebae [242]. Several measures to control and eradicate biofilm formation and development can be implemented, such as nutrient control, contamination control of materials and equipment, cross-connection control and backflow prevention, disinfectant residuals, and corrosion control [195].

Recently, the frequency and diversity of NTM in hospital soil and dust of Iraq was analyzed, with 8.8% of the samples being positive and harboring 33 species of mycobacteria (19 rapidly growing and 14 slowly growing species), with *M. setense* and *M. lentiflavum* as the most prevalent species [204].

## 5. Mycobacteria Infection

The opportunistic pathogenicity of NTM has been the focus of research in humans, livestock, wildlife, and laboratory model species such as zebrafish. In humans, the treatment of diseases caused by NTM infection is particularly challenging, due to its long duration, variability in bacterial susceptibility, and lack of evidence-based guidelines. Treatment usually consists of a combination of at least three drugs administered during months to years, often leading to severe secondary effects and a high chance of relapse in addition to high costs.

### 5.1. Human Infection

*Mycobacterium* species can be classified as strict or opportunistic pathogens, according to the relationship established with the host [12]. The research interest and focus in the *Mycobacterium* field has been centered on two of the most clinically relevant strict pathogens for humans, *M. tuberculosis* and *M. leprae*. 

Whilst unprecedented changes occur worldwide, populations aging and the underlying development of chronic conditions thread the ground for the increasing prominence of NTM as opportunistic pathogens. Most contacts with NTM in humans are transient and self-curing colonization, with the immune system being able to clear the bacilli in most immunocompetent patients, however in individuals with risk factors an infection might be established. Several routes of infection have been proposed including (1) aerosolization and inhalation, (2) swallowing and aspiration, and (3) introduction into wounds.

The main source of nosocomial infection is water sources due to their low total microbial burden, leading to a proliferation of resistant and oligotrophic bacteria, among them NTM, together with the direct contact with the oronasal cavity and the possibility of aerosolization [243]. For that reason, most cases of NTM infection in humans have been reported due to contact with hot-tub use [183], showering [31], faucet use [244], gardening [240], commercial farming [245], aerosolized metalworking fluids [172], and tending of aquariums [246]. However, the existence of huge lag time (of 1 to 10 years) between estimated colonization and diagnosis of the disease makes it very difficult to accurately pinpoint the specific source [138]. In addition, inhalation, ingestion, and inoculation from environmental sources are also shown routes of transmission. There is no established evidence of human-to-human transmission. The slow-growing strains of NTM possess a higher potential to become pathogenic due to their close taxonomic relatedness to obligate pathogens that colinks with biological traits endorsing resilience to the host environment [247].

According to the available data, the incidence of NTM disease varies considerably with NTM species, geographic distribution, sex, race/ethnicity, age, and risk factors (e.g., comorbidities). While initial reports described older male patients with predisposing conditions, nowadays nearly 80% of patients are middle-aged or elderly females [176]. Pulmonary disease caused by NTM usually occurs in individuals that are sensitized by other lung-associated diseases, such as chronic obstructive pulmonary disease (COPD), changes in lung and chest architecture, α-1-antitrypsin deficiency, cystic fibrosis, heterozygosity for cystic fibrosis transmembrane conductance regulator (CFTR) mutations, gastric reflux disease, rheumatoid arthritis, and immunodeficiency or immunosuppression due to human immunodeficiency virus (HIV) infection, organ transplant, cancer, or chemotherapy [73]. Additionally, increased susceptibility of human hosts to NTM has been observed due to mutations in five genes (*IFNGR1*, *IFNGR2*, *IL12RB1*, *IL12B*, and *STAT1*) [195].

The major manifestations of NTM infection are pulmonary, in the lymphatic system, osteoarticular, and skin and soft tissue [240]. Disseminated disease via hematogenous spread has also been described. Pulmonary infections are the most common, originating from aerosolized particles from water or soil sources, with a clinical presentation similar to TB [248]. Regarding extra-pulmonary infections, localized lymphadenitis (enlargement of one or more lymph nodes resulting from inflammation), is common in children between 1 and 5 years old and mostly caused by MAC, *M. scrofulaceum*, *M. malmoense*, and *M. hemophilum* [249]. Osteoarticular infections are rare since their development is usually driven by inoculation of the NTM for contiguous infection due to surgical procedures, penetrating trauma, injuries, or needle injections [250]. The most frequent NTM isolated in these cases are *M. chelonae*, *M. hemophilum*, *M. marinum*, and *M. kansasii* [250]. Skin and soft tissue infections occur after traumatic injury, surgery, or cosmetic procedures, with wound exposure to the soil, water, or contaminated prosthetic or other medical devices [251]. Disseminated disease occurs in individuals with severely compromised immune systems, especially in patients infected with HIV. 

Antibiotics are the main therapy treatment of NTM diseases, however, each NTM species and each patient demands different antibiotic combinations, leading to prescription difficulties, due to limitations of in vitro susceptibility tests to correctly predict MIC concentrations [138]. The basis of all current treatments for NTM infections is macrolides. These antibiotics have the best correlation between in vitro susceptibility results and clinical (in vivo) response. Approaches to multidrug therapy of pulmonary NTM disease, as summarized in the latest American Thoracic Society/Infectious Diseases Society of America guidelines, involve seven basic concepts [240]: (1) macrolide-based regimens are suggested for the treatment of infections caused by most slowly growing NTM; (2) ethambutol is usually added to multidrug regimens as an ‘enhancing’ drug; (3) either rifampin or rifabutin is usually added as a third drug in a multidrug regimen; (4) ancillary treatment on a case-by-case basis of aminoglycoside therapy; (5) decision on whether a daily therapy or an intermittent therapy; (6) therapy duration of at least 12 months beyond sputum culture conversion to negative; (7) infection by rapidly growing NTM are very difficult to treat, with a high rate of relapse. Extra-pulmonary infection therapy is usually based on surgical removal of the infected lymph node or infected area and/or antibiotic therapy similar to that applied to pulmonary NTM infections [240]. The Clinical and Laboratory Standards Institute had recommended the broth microdilution method as the gold standard for antimicrobial susceptibility testing of NTM [252].

#### Clinically-Relevant Species

Due to their environmental nature, NTM mycobacteria are distributed worldwide, however, several knowledge gaps remain, namely concerning disease incidence across countries and continents, as disease notification is not mandatory. A prevalence report of NTM in pulmonary samples evidenced a differential distribution, with MAC, *M. xenopi*, *M. fortuitum*, and *M. abcessus* dispersed throughout the globe, and *M. kansassii* and *M. malmoense* with a higher incidence in Europe [180]. Overall, *M. ulcerans*, MAC, *M. kansasii*, *M. malmoense*, *M. abscessus*, *M. chelonae*, *M. fortuitum, M. xenopi*, and *M. marinum* are globally the most clinically relevant groups.

*M. ulcerans* causes the third most common mycobacterial disease in the world, the Buruli ulcer, following TB and leprosy, with a total of 2713 cases reported in 2018, mainly in countries from West and Central Africa [253]. *M. ulcerans* affects mainly skin and bone, and the clinical presentation can vary from a localized nodule or ulcer to generalized ulcerative or nonulcerative disease, leading to long-term disability [252]. The transmission pathway to humans is unknown, however, evidence suggests that mild skin injury after exposure to contaminated environmental sources, such as water or soil, can be a transmission route [252].

MAC currently encompasses 14 members, being *M. avium* subspecies *avium* (MAA), MAP, MAH, *M. intracellulare*, and *M. chimaera* the most clinically relevant members [254]. MAC can survive in several environmental conditions, isolated from water systems in residences and hospitals, and is the most common NTM associated with lung disease worldwide [255]. Cutaneous involvement of MAC infections has been rarely reported, with the infection been a result of trauma, cosmetic procedures, and postsurgical infections [251]. Several reports indicate a tendency to increase the prevalence of MAC species in the USA, Europe, and South Korea [256]. MAA is also connected to lymphadenitis in young children; and *M. chimaera* was recently implicated in disease in patients subjected to cardiac surgery [257]. MAP causes a chronic granulomatous enteric infection in ruminants, mainly cattle, named Johne’s disease, and has a long-term hypothesized association with Crohn’s disease in humans.

*M. kansasii* is responsible for pulmonary disease with nodular bronchiectasis and fibrocavitary forms of clinical presentation, being the second most common cause of NTM lung disease in some European countries and the USA [255,258]. However, individuals with extra-pulmonary and disseminated disease were already reported [258]. Patients with previous lung conditions like COPD, bronchiectasis, or tumors are group risks [258]. Furthermore, in immunocompromised patients with HIV infection or subjected to renal transplantation, *M. kansasii* can upsurge as a cutaneous infection as well, sometimes in a concomitant way, or even as a disseminated disease episode [258]. The tap water is pointed out as the main reservoir of *M. kansasii* and the contamination occurs through aerosol inhalation [252,258]. However, despite this species being considered as an environmental opportunistic pathogen, the strains associated with human infections have not yet been isolated from environmental samples, which suggests a different source of infection [258].

*M. malmoense* is responsible for causing pulmonary disease with a clinical presentation similar to TB [259]. A few reports of lymphadenitis in children and tenosynovitis by *M. malmoense* were reported by the medical community [252]. The isolation from water and soil is reported in the literature, however, the environmental sources are not well defined. *M. malmoense* infection is frequently reported in the UK and northern Europe [176].

Nowadays, genomic data support the classification of *M. abscessus* in *M. abscessus* subsp. *abscessus*, *M. abscessus* subsp. *bolletii*, and *M. abscessus* subsp. *massiliense* [260]. These mycobacteria can be found in several environmental matrices including water, soil, and dust [260]. This species is one of the most frequently notorious NTM species accountable for severe respiratory, skin, and mucosal infections in humans, being one of the most antibiotic-resistant NTM, leaving few therapeutical options to be implemented [261]. Infections associated with surgical or aesthetic contaminated equipment are also reported [262,263]. The study of outbreak clusters suggests the possibility of human-to-human transmission although definitive evidence has not been provided [264]. The differentiation into the subspecies level is important since it enables disease prognosis due to distinctions in resistance to treatment [260,261]. 

*M. chelonae* was already isolated from tap water, freshwater, and seawater [252]. It can cause infections in the skin, soft tissues, and bones, and was previously related to infected piercing wounds, contaminated tattoo inks, plastic surgery, or liposuction [252]. Moreover, it is usually found in hospital settings [256]. These species are extremely resistant to antibiotics and disinfectants, being major nosocomial pathogens [256].

*M. fortuitum* is an environmental mycobacterium and is included in a complex with nine others. Similarly to *M. chelonae*, it is also associated with skin, soft tissue, and bone infections. *M. fortuitum* was previously associated with plastic and cardiac surgery interventions [262,263].

Infections by *M. xenopi* affect essentially the lung, with a clinical presentation of pulmonary cavities and nodules, however cases with the involvement of soft tissues were previously reported [252]. Similar to other NTM, immunocompromised individuals or people with pre-existent pulmonary conditions are risk groups [252]. *M. xenopi* is the third most common mycobacterium isolated from pulmonary specimens in Europe [180]. It is mainly recovered from warm tap water, and it was already recovered from hot water systems in hospitals and surgical utensils as bronchoscopes.

*M. marinum* is an important fish pathogen causing the development of granulomatous lesions in fresh and marine water fish [265,266]. The punctures from the fins of fish or shrimp in humans can cause ulcers and nodules in soft tissue located in fingers, hands, arms, elbows, knees, or toes; and granulomatous lesions in deep tissues [252,265]. The transmission of *M. marinum* could also occur by cleaning of fish tanks or fish processing industry [252,265]. In rare cases, deep tissue infection or disseminated disease can occur in immunocompromised patients [265].

In addition to direct disease caused by NTM species, several diseases are cryptically related to NTM contact: chronic bowel disease, allergies, and pulmonary viral infections. Chronic bowel disease, also known as Crohn’s disease, has been proposed to be associated with MAP infection, also driven by the observation of patient improvement after antimycobacterial therapy, however, the presence of this species has seldom been detected in affected patients, so this topic possesses great controversy [267,268]. In addition to MAP infection, *M. tuberculosis* [269] and *M. genavense* [270] intestinal infections have been reported. 

Paradoxically, both the increase and the reduction of human contact with NTM species have been reported to increase allergies. The increase of human contact with NTM can lead to the development of hypersensitivity pneumonitis, an inflammation of the lungs caused by the release of inflammatory byproducts of mycobacteria; contrarily, the reduction of human contact with NTM can lead to a shift from Th1 to Th2 immune responses, promoting allergies [271,272]. Pulmonary viral infections are strongly associated with a dysregulation of pulmonary immunity, similarly to what happens with hypersensitivity pneumonitis, which can increase the predisposition to be infected by aerial viruses [76].

The multifaceted effects of NTM interactions with humans resulting in augmented or diminished immunity to TB and leprosy are not yet understood. Serious issues of cross-reactivity between human NTM exposure and intradermal skin testing have been extensively studied over the years, with individuals colonized with NTM that do not develop disease, inducing a non-specific immune response to the Mantoux test and leading to false-positive reactions in tuberculin testing [273,274]. 

Therefore, depending on the timing, dosage, bacterial state, and route of exposure, NTM can prevent or predispose to a variety of clinical situations [273,274]. Moreover, previous exposure to NTM, including MAC members, have been shown to reduce BCG vaccine efficacy against TB in humans [275]. Worldwide, BCG efficacy decreases with latitude, which has been hypothesized to be related to an increase in NTM abundance [275].

The countries best represented in the literature have a lower prevalence of NTM infection, better diagnostics for diseases associated with NTM, and more funding available for research [138]. The imbalance in material and human resources across countries or continents difficult the assessment of the role of climate and geographical location in the exposure of susceptible people to NTM from water and soil [138]. Unfortunately, official figures of infections due to NTM cannot be given because they are mostly not incorporated in surveillance programs. In developing countries, it may be difficult to assess the prevalence of NTM infections, mainly because identification of the species involved is generally not done and these diseases are often under-diagnosed, diagnosed with considerable delay, or misdiagnosed as TB. The absence of information regarding the burden of NTM results in inappropriate management and poses significant challenges for infection control strategies, which can lead to serious morbidity and mortality [256].

### 5.2. Animal Infection

Regarding animal infections, there are similarities with the human counterpart, with mammals developing persistent cutaneous or subcutaneous nodules, fistulous draining tracts, and granulomas in soft tissues, mostly with localized disease [169,276]. NTM infections seem to be mainly digestive, with primordial manifestation in intestinal mucosa and mesenteric lymph nodes, however, cutaneous and respiratory infection routes are also possible, namely by retro-bronchial and pulmonary routes, mainly due to dust inhalation during grazing in stabulation [169]. There is no proof of animal-to-animal transmission. Several NTM species can be found in the microbiota of animals, namely rapid-growing NTM, with disease occurring opportunistically [169,276].

Among the different species of NTM previously reported causing disease in animals are MAC, *M. kansasii*, *M. marinum*, *M. xenopi*, *M. chelonae*, *M. abscessus*, *M. fortuitum*, *M. smegmatis*, *M. phlei*, *M. porcinum*, *M. farcinogenes*, *M. senegalense*, and *M. scrofulaceum* [169,276].

Several MAC members have been reported to cause infection in livestock and wildlife species, for instance, dogs from the Basset hound dog breed that may develop systemic *M. avium* disease [276]. MAC infection has been reported as the most common disseminated bacterial infection in rhesus macaques with the simian immunodeficiency virus [277]. *M. intracellulare* has been identified in a rusty monitor reptile with lung nodules and *M. avium* was isolated from cockroaches from a South Taiwan hospital [278,279]. In addition, several wild animals can become infected with MAC species, namely ruminants, lagomorphs, rodents, ungulates, carnivores, and several raptor species [280]. MAA is the main etiological agent of avian TB. Regarding MAP, the most commonly detected MAC member, several reports describe infection in cattle, sheep, and goat, as well as in wild ruminants, including red deer, roe deer, fallow deer, white-tailed deer, alpine ibex, and riverine buffalo. In non-ruminant animals, MAP was first detected in wild rabbits, brown bear, raccoon, opossum, coyote, red fox, stoat, weasel, wood mouse, European badger, European brown hare, jackdaw, and Egyptian mongoose, which may be exposed via feeding on contaminated grain, forage in pastures, feces, or predating on infected prey [280]. Transmission between wildlife and livestock occurs via fecal contamination of feed and forage, both in farm buildings and on pastures [281]. Once in the environment, MAP can survive for several years, being washed away by rainfall from contaminated pastures into rivers [282]. Published works suggest higher prevalence of MAP in Europe and North America, which also possible results from more research efforts [282].

The presence of *M. kansasii* has been rarely reported from asymptomatic ungulates, pigs, dogs, and squirrel monkeys [283,284,285,286]; from respiratory tract lymph nodes in cattle; and isolated from skin tissue as well [169]. *M. kansasii* infection resulting in inflamed lymph nodes or pneumonic lesions has also been described in monkeys, llamas, goats, and domestic and feral pigs [285,287,288,289]. Additionally, this bacterium has also been isolated from unpasteurized cow milk [290].

*M. marinum* is mainly found in cultured, ornamental, and wild fish living in densely populated habitats, with water temperature and water quality being essential for infection development. Examples of commonly infected fish species include salmon, striped bass, turbot, tilapia, zebrafish, rabbitfish, and sturgeon [291,292,293,294,295,296,297]. The most commonly reported sources of infection in production fish are contaminated fish pellets produced from fish products. In addition to fish, several domestic animals and several wild animals (manatee, European hedgehog, bullfrog, and American toads) are reported as susceptible to *M. marinum* infection [298,299,300,301].

*M. xenopi* is mainly isolated from the mesenteric lymph nodes of pigs with tuberculosis-like lesions, but also from amphibians, birds, felines, and ungulates in which water is suspected as the main infection route [302,303,304,305,306].

*M. chelonae* is responsible for several reported infections in aquatic species, such as disseminated granulomatosis in fish, TB-like lesions in turtles’ lungs, and abscesses and ulcers in manatees [276]. Additionally, several abscesses or lesions in lymph nodes and other tissues have been reported in several mammalian species, however, there is a possibility of those cases having been caused by *M. abscessus*, that was posteriorly designated as a separate species [276].

*M. fortuitum* causes disease in several mammals, namely granulomatosis in skin, lungs, lymph nodes, and joints [276]. Dogs of the Doberman pinscher breed have been hypothesized as more susceptible to disseminated mycobacterial infection [276]. *M. fortuitum* is also associated with infections in wild animals including reptiles, amphibians, invertebrates, ungulates, and aquatic mammals [307,308,309]. 

*M. smegmatis* have been causative agents of granulomatous mastitis in cattle, ulcerative skin in cats, systemic infections in dogs (Basset hound breed) with granulomatous lesions found across the liver and lymph nodes [276]. Cats can also develop rare ulcerative skin lesions from *M. phlei* infection [276].

*M. porcinum* is a rapidly-growing NTM, almost exclusively reported as a non-human opportunist pathogen and the causative agent of bovine farcy [310]. This disease is characterized by chronic granulomatosis in cattle, mainly in tropical areas [310]. In addition, *M. porcinum*, *M. farcinogenes*, and *M. senegalense* can also be the causative agent of bovine farcy [310]. *M. porcinum* can cause lymphadenitis in pigs [276].

*M. scrofulaceum* has been isolated all over the world from ungulates, swine, primates, fish [306,311,312], and cattle and mice feces, with a putative risk of contamination via the fecal-oral route [313] and from diptera members that could act as disease vectors in cattle and pig farms [314].

Moreover, co-infection with members from the MTC and MAC or other NTM species is not rare in animal and human hosts [203,315]. Wildlife and domestic animals may develop TB-like lesions due to mixed infections of *Corynebacterium pseudotuberculosis* and NTM [316], making these animals a permanent reservoir of NTM and a threat to control and eradication programs [203].

Non-specific sensitization of cattle to bovine tuberculin and IFN-γ may result from exposure to MAC species or other NTM, such as *M. hiberniae*, *M. cookii*, *M. fortuitum*, or MAP [169]. When these non-specific reactions are common, the use of MTC specific antigens, mainly in the case of low bovine TB prevalence areas, is highly recommended [169]. In addition, the single intradermal comparative tuberculin test enables the assessment and comparison of delayed immunity reactions to avian and bovine tuberculin in parallel. Mixed infections with MAC species and *M. bovis* have been identified as factors leading to false-negative results using both tuberculin and the IFN-γ test [169].

## 6. Detection, Identification, and Differentiation Tools for Mycobacteria

Environmental matrices are mostly studied in the context of NTM infection for which person-to-person transmission is very difficult to demonstrate. Both culture-dependent and independent approaches rely on the intrinsic characteristics of the type of matrices tested. The isolation of mycobacteria, namely the slow-growing, is very difficult from microbially rich environments, for which harsh decontamination is recommended [317]. In addition, the intrinsic characteristics of several species of *Mycobacterium* require specific micronutrients (e.g., *M. haemophilum*), growth enhancers (e.g., *M. genavense*), optimal pH, temperature, and oxygen levels, which allied to a particular sensibility to decontamination (e.g., *M. ulcerans*) sets obstacles to bacteriological isolation [252,318]. 

The composition of environmental samples is complex and influences the optimal application of the techniques. For water samples, both from natural water sources (e.g., surface waters or rivers) or anthropogenically-derived (e.g., drinking water systems or household water), most authors concentrate large volumes of water by filtration and/centrifugation [114,319]. Membrane filtration is standardized. This membrane can be used for mycobacterial culture, however, the ratio of mycobacteria to other microorganisms in water samples is usually low, requiring decontamination for better performance, normally with cetylpyridinium chloride (CPC) [82]. CPC has been tested widely in different water samples and even in mycobacterial biofilms, being indicated as the most efficient decontaminant for such samples.

For soil samples, the biogeochemical composition of each type of soil (e.g., pH, ionic charge, presence of particulate matter) influences greatly the success of culturing mycobacteria and DNA extraction efficiency. Other intrinsic characteristics of mycobacteria, like the ability to adhere to soil particles further hamper isolation during bacteriological culture. Soil samples also present inhibitors (e.g., humic acids) that need to be eliminated to achieve mycobacteria DNA amplification from soil nucleic acid extracts. Several decontaminants have also been tested, but all of them seem to reduce the number of mycobacteria isolated [252]. Efforts have been made to improve the selective potential of media to isolate mycobacteria from soil samples, e.g., by adding 250 mg/L malachite green to 7H10 agar [320]. Additionally, cell detachment from soil particles can be accomplished using chemical and/or physical processes. Different classes of chemical reagents can be used for cell detachment, from non-ionic surfactants to salts and ionic dispersants [321]. These chemical reagents usually work by weakening intermolecular forces that attach the cell to the substrate [321]. Physical detaching can be more often accomplished by vortex, sonication, and ultrasounds [321]. These physical methods release bacteria that are entrapped in micropores or channels [321]. Standardized methods for isolation of mycobacteria from environmental matrices have thus been difficult to achieve.

Phenotypic methods are still the most suited methods for the identification of mycobacteria in low-income countries, where often trained staff is scarce and/or uneven access to molecular biology techniques. In a review by Bhalla and co-workers (2018), a set of 13 parallel phenotypic tests enables the identification of the most frequently isolated species of NTM, including niacin accumulation, arylsulfatase, nitrate reduction, thermostable, high catalase, low catalase, hydrolysis of Tween-80, citrate utilization, iron uptake, urea hydrolysis, growth in presence of 5% NaCl, growth in MacConkey agar without crystal violet, and tellurite reduction tests [322]. In a work by Khosravi and coworkers (2017), a set of ten phenotypical assays enabled the identification of 89.7% of 98 clinical isolates, when compared to *rpoB* sequence analysis [323].

Another culture-dependent method that has been applied to the identification of mycobacteria is matrix-assisted laser desorption/ionization mass spectrometry (MALDI-TOF MS). For this purpose, bacterial extracts or intact microorganisms are used which, in the particular case of mycobacteria, require the inactivation and disruption of the mycolic acid-rich cell wall [324]. Those molecules are ionized, transferred into a gas phase and the resulting ions are separated according to their mass/ charge ratio [324]. Such extracts produce different mass spectra that can be used for identification [324]. This technique was initially developed targeting the identification of members of MTC for the clinical approach but has more recently been expanded to the identification of NTM [324,325,326], while databases for spectra analyses are growing.

The mycobacterial cell wall is a critical structure for mycobacteria that has also been exploited for diagnostic purposes. Multiple chromatographic techniques have been employed since the 1980s aiming the analysis of mycolic acids to identify patterns characteristic of each *Mycobacterium* species [327,328,329], switching from thin-layer chromatography [327,328] to high-performance liquid chromatography (HPLC) [329].

Fluorescence in situ hybridization (FISH) assays designed for mycobacteria target mostly the 16S rRNA or Internal Transcribed Spacer (ITS) genes with both DNA and peptide nucleic acid (PNA) probes [330]. Most reported FISH assays target MTC and/or NTM in the context of clinical specimens from sputum samples [330]. The use of the MN Genus-MTBC FISH commercial kit, which contains a probe for an internal 16S rRNA sequence that the *Mycobacterium* genus shares with *Nocardia* genus, and an MTC-specific probe that is complementary to a 23S rRNA 5’ sequence, has been evaluated. Shah and coworkers (2017) reported the correct identification of 44 reference *Mycobacterium* species using this approach, except for *M. wolinskyi*, confirming that the panel of six strains belonging to the MTC specifically reacted to the MTC-specific probe [331]. In the same work, while testing the MTBC-MAC FISH commercial kit, only the six MTC members among the 44 reference species reacted to the MTC-specific probe, and the 14 reference MAC members reacted to MAC-specific probe [331]. PNA probes have been developed for MTC members as well as for NTM, in which a sensitivity of 71.4% and specificity of 100% was obtained in clinical specimens [332]. In another study, a specific PNA probe targeting the 16S rRNA of MAA hybridized with MAP besides its initial target, being successful in MAA identification in both biofilm and potable-water samples, however, the struggle around background fluorescence was also reported [333].

Most emerging techniques (e.g., MALDI-TOF MS, FISH, or flow cytometry) are culture-dependent and do not minimize mycobacteria isolation issues. Flow cytometry is a widely used technique in the context of physiology and immune response studies, usually not focused on environmental matrices which present an inherent complexity that barrier the development of protocols for detection and identification of mycobacteria. It has been applied to mycobacteria, essentially restricted to research works mostly focused on the immune response [334], antibiotic resistance, and physiology [335]. By using high specificity probes, similar to those used for FISH, it could provide the advantage to simultaneously identify *Mycobacterium* species and, if fixation processes are not used, then it might provide the opportunity to evaluate physiological parameters, like cell wall integrity or metabolic activity. Moreover, the FISH technique coupled with flow cytometry allows a high-throughput characterization on a single-cell level based on the fluorescence resulting from in situ hybridization, allowing the combination of both taxonomic identification and rapid and correct quantification of microbial communities. Furthermore, cells can be sorted after flow cytometry analysis with several possible downstream applications, ranging from omics (e.g., genomics), cultivation, antimicrobial sensitivity testing, among other processes, enabling a better understanding of the NTM role in the environmental community, the viability analysis of NTM community, etc. Kamariza and coworkers (2018) reported that *M. smegmatis* with 4-N,N-dimethylamino-1,8-naphthalimide–conjugated trehalose (DMN-Tre) labeling exhibits increased fluorescence over the background due to metabolic conversion within the mycomembrane, in sputum samples [336]. However, particularly in soil samples, methods like FISH and flow cytometry that depend on the hybridization of probes and fluorescence detection face additional challenges. Many times, particulate matter follows the processing of samples along with bacteria and reaches the endpoint. Those particles influence the optical acquisition and may present background fluorescence introducing confounding effects, as well as, diminishing the hybridization efficiency of the probe. In addition, the downsides of such techniques is their cost and the need of technical expertise.

In addition to the culture-dependent approach, which requires the long-lasting process of isolation of mycobacteria, there is a wide variety of molecular techniques available for the detection and identification of NTM species. One of the most commonly applied techniques for bacterial identification is 16S rRNA gene sequence analysis. Conserved or variable regions of the 16S rDNA amplicon are amplified depending on the goal of detection or identification. Still, due to the high similarity found across mycobacterial sequences, this analysis is frequently coupled with *rpoB*, associated with rifampicin resistance, and also by analysis of *hsp65* that together enhance discriminatory power [138,337]. The single-use of the 16S rRNA gene may lead to misidentification of species, contrary to *rpoB* sequencing which seems to achieve better resolution at the species level [338]. The use of *rpoB* sequencing for identification is wider in NTM, including MAC and RGM [338], and enables identification of approximately 80% of isolates from these species to species level [338]. The attempt to establish a reliable identification database has been the target of many efforts over the last years to fulfill one of the major gaps in the application of this multi-locus approach [337]. In the context of drinking waters, the *rpoB* sequencing was capable of successfully identifying approximately three thousand sequences per water sample, allowing the comparison of water with different ages [339].

Insertion sequences (IS) have been widely used for the identification of mycobacteria. Several methods of both conventional and real-time PCR have been developed based on those sequences. Within the MAC, several subspecies can be identified by the presence of IS, namely, *IS901* in MAA [340] and *IS902* in *M. avium* subsp. *Silvaticum* [341], amongst other ISs that enable differentiation besides identification. In the case of *M. ulcerans*, identification is possible through analysis of *IS2404* and *IS2606* [342]. Some other species have been described to have IS useful for identification purposes, like *IS1407* in *M. chelatum* [343], *IS1395* in *M. xenopi* [344], *IS1511/1512* in *M. gordonae* [345], *IS1652* in *M. kansasii* [346], and *IS6120* in *M. smegmatis* [347].

More recently, Sevilla et al. (2015) proposed a novel tetraplex real-time PCR that permits identification of *Mycobacterium* genus by ITS amplification, as well as identification as *M. avium* by *IS1311* amplification and/or as MTC by *devR* amplification, coupled with an internal amplification control [315]. This protocol was initially tested for animal samples and milk but has great potential for the identification of mycobacteria in environmental samples [315].

DNA probe commercial tests are also widely used for the identification of NTM. Amongst those tests is INNO-LiPA MYCOBACTERIA v2 which is a reverse hybridization DNA probe assay capable of identifying 16 different species of mycobacteria [348]. Three of the probes included in the assay were found to cross-hybridize with some isolates rarely found in clinical samples [348]. More recently, it was also reported the misidentification of *M. smegmatis* for *M. fortuitum* complex in a case study event [349]. Another similar test is GenoType NTM-DR which is capable of identification of the more relevant clinical NTM species, as well as detection of resistance to both macrolides and aminoglycosides [350]. In recent years, this assay has been tested for identification of MAC and *M. abscessus* with 92% accuracy for reference strains and 100% for clinical isolates, though three MAC reference strains were misidentified as *M. intracellulare* [350]. In another study with MAC isolates, 91% of isolates were identified correctly to the species level [351].

Regarding the differentiation of NTM, most techniques have been adapted from MTC differentiation protocols. For most NTM, the gold standard for differentiation still is Pulsed-Field Gel Electrophoresis (PFGE). This technique has high costs and requires technical expertise, with a long-lasting protocol that put it aside for clinical settings, but still one the most discriminatory for NTM [352]. PFGE was applied to several NTM species (e.g., *M. kansasii* [353], MAC [354], *M. gordonae* [355], *M. haemophilum* [356]), with relative success.

Another technique that appears to achieve good results in NTM differentiation is Random Amplified Polymorphic DNA (RAPD) analysis. One of its low points is the fact that differences in technical applications may influence reproducibility [352]. However, it remains useful for NTM differentiation and it was used in species like *M. abscessus* [357], *M. mucogenicum* [186], *M. phocaicum* [186], and *M. gordonae* [358]. In the particular case of *M. avium*, the differentiation has been based on the restriction fragment length polymorphism (RFLP) typing of *IS1245*, which appears as reference for this species [359]. 

A promising tool in the range of molecular techniques is whole-genome sequencing (WGS). In addition to the ability for extensive species characterization and categorization that is particularly relevant for the *Mycobacterium* genus, WGS enables the discovery of novel biomarkers helpful in the context of pathogen identification [17]. Recently, it was proposed the use of the *ku* gene as a biomarker for *Mycobacterium* genus [360]. In that study, 79.7% of all known mycobacterial species and all submitted genomes were considered for comparative genomics, indicating that this gene is widely distributed and conserved across *Mycobacterium* species [360]. Gupta and coworkers (2018) indicate that ten conserved signature indels and nine conserved signature proteins are shared by most members of the *Mycobacterium* genus [13]. Those biomarkers have the potential to be used as targets in PCR-based methods, Enzyme-Linked Immunosorbent Assay (ELISA), etc. Although WGS requires DNA purity and quantity, which is sometimes harder to achieve while working with environmental samples, it allows answering to both identification and differentiation questions. Small quantities of DNA can be overcome using whole-genome amplification techniques. In addition to those fundamental elements for isolate identification and differentiation, WGS can also provide insights into antibiotic resistance patterns in environmental isolates, epidemiological issues, etc., that may be relevant in the clinical context.

A long-lasting discussion on the pros and cons of culture-dependent and culture-independent approaches remains. The main advantage of the culture-dependent approach consists of the detection of viable mycobacteria (colony formation) within the clinical sample, which increases the probability of these being the causative disease agent. However, the time needed for mycobacteria cultivation can be high with some species (e.g., MAP) being extremely fastidious. Additionally, the steps necessary to eliminate other bacteria from the sample (decontamination step) can cause mycobacteria fragility and lead to culture unviability and false negative results. On the other hand, culture-independent approaches, such as those enabled by PCR, present fastness as their main advantage. These approaches, however, do not enable the assessment of cell viability, leading to uncertainties regarding causation between DNA presence of a bacterium and disease. Technologies like flow cytometry coupled with FISH are promising approaches to taxonomically identify bacteria present in a sample, together with their viability status, in a high-throughput manner.

## 7. Conclusions

The NTM background as environmental bacteria enables mycobacteria persistence in a gradient of ecological niches, from natural to anthropogenic environments, posing challenges to the identification and differentiation of these opportunistic pathogens when specifically involved in clinical settings and, consequently, to the adoption of the most effective control and treatment strategies. The identification and differentiation of NTM is a growing research field, with MALDI-Tof, FISH, flow cytometry, and WGS progressively assuming a steady relevance. 

The striking ecology of NTM results from the combination of several biological features, namely slow growth, a hydrophobic and lipid-rich impermeable cell wall, genetic variability associated with plasmid-mediated horizontal gene transfer and recombination, and transcriptional regulation signatures. Biofilm formation ability, symbiosis with protozoa, resistance to extreme pH stress, survival under anoxic or anaerobic conditions, and a remarkable metabolic activity of recalcitrant carbon compounds, also contribute to the adaptation and persistence of NTM in a huge variety of environments.

NTM represent an increasing concern in the medical field, emerging as opportunistic pathogens, especially in immunocompromised and elderly individuals, with *M. ulcerans*, MAC, *M. kansasii*, *M. malmoense*, *M. abscessus*, *M. chelonae*, *M. fortuitum*, *M. xenopi*, and *M. marinum* representing the most clinically relevant species. This increased concern results not only by the increasing number of infections but also for the NTM natural and acquired capacity to survive antimycobacterial therapy. Moreover, NTM are also disease agents for domestic, livestock, and wildlife animals, raising apprehension related to their putative zoonotic potential. Alternative, non-conventional therapies have thus become hotspots of investigation.

This review gathers up to date biological and ecological information of NTM, identifying knowledge marks but also research gaps that need to be addressed, stressing out that decision-makers need to recognize NTM as a public health concern that needs to be tackled in an increasingly susceptible elderly and immunocompromised population, as well as in low- or middle-income countries, where NTM infections are still highly misdiagnosed as tuberculosis. Antimicrobial resistance of NTM to several drugs limits therapeutic options and showcases the need for research investment in alternative approaches.
